# Chikungunya Death Risk Factors in Brazil, in 2017: A case-control study

**DOI:** 10.1371/journal.pone.0260939

**Published:** 2022-04-07

**Authors:** Rhaquel de Morais Alves Barbosa Oliveira, Francisca Kalline de Almeida Barreto, Geovana Praça Pinto, Isabella Timbó Queiroz, Fernanda Montenegro de Carvalho Araújo, Kilma Wanderley Lopes, Regina Lúcia Sousa do Vale, Daniele Rocha Queiroz Lemos, John Washington Cavalcante, André Machado Siqueira, Lívia Carla Vinhal Frutuoso, Elisabeth Carmen Duarte, Antônio Silva Lima Neto, André Ricardo Ribas Freitas, Luciano Pamplona de Góes Cavalcanti

**Affiliations:** 1 Programa de Pós-graduação em Saúde Coletiva, Universidade Federal do Ceará, Fortaleza, CE, Brasil; 2 Faculdade de Medicina, Centro Universitário Christus, Fortaleza, CE, Brasil; 3 Laboratório Central de Saúde Pública do Ceará, Fortaleza, CE, Brasil; 4 Secretaria Municipal de Saúde de Fortaleza, Fortaleza, CE, Brasil; 5 Programa de Pós-graduação em Patologia, Universidade Federal do Ceará, Fortaleza, CE, Brasil; 6 Serviço de Verificação de Óbitos Dr Rocha Furtado, Secretaria de Saúde do Estado do Ceará, Fortaleza, CE, Brasil; 7 Instituto Nacional de Doenças Infecciosas da Fundação Oswaldo Cruz, Rio de Janeiro, Brazil; 8 Universidade de Brasília, Programa de Pós-Graduação em Medicina Tropical, Brasília, DF, Brasil; 9 Ministério da Saúde, Secretaria de Vigilância em Saúde, Brasília, DF, Brasil; 10 Faculdade São Leopoldo Mandic de Campinas, Instituto de Pesquisa São Leopoldo Mandic, Campinas, Brasil; University of Ibadan, NIGERIA

## Abstract

**Background:**

In 2016/2017 we had a major epidemic of chikungunya (CHIK) in Brazil, with many deaths. We evaluated to factors associated with deaths from CHIK that occurred in the city of Fortaleza, Brazil.

**Methods:**

A matched case-control study was conducted (1:2), by sex, age (± 5 years) and neighborhood. Cases were CHIK deaths that occurred between January 1 and December 31, 2017, in Fortaleza, Brazil, and which were laboratory confirmed. Controls were laboratory confirmed CHIK patients occurring in the same neighborhood and in the same period, but which did not progress to death.

**Results:**

82 cases of CHIK and 164 controls were included. Considering the clinical history, significant associations were found between other chronic heart diseases (OR 3.8; CI: 1.53–9.26) and chronic kidney disease (OR 12.77; CI: 2.75–59.4). In the multivariate analysis of the variables related to signs and symptoms, fever (OR: 19.23 CI: 1.73–213.78), abdominal pain (OR: 3; 74 CI: 1.06–13.16), apathy (OR: 11.62 CI: 2.95–45.82) and dyspnea (OR: 50.61; CI: 12.37–207.18) were identified with greater likelihood of death from CHIK. It also stood out that altered blood glucose was associated with cases with a worse prognosis (OR: 13.5; CI: 1.3–135.0). Among the laboratory findings, only lymphocytes and albumin were not associated with greater likelihood of death.

**Conclusion:**

The factors related with deaths were chronic kidney disease and previous heart disease, presence of fever, abdominal pain, apathy, dyspnea and arthritis and laboratory findings such as leukocytosis, leukopenia, thrombocytopenia, neutropenia and lymphopenia.

## Introduction

The chikungunya virus (CHIKV) is an alphavirus, belonging to the Togaravidae family, which was described in 1952 in Tanzania [1]. Chikungunya (CHIK) presents a clinical picture that can vary from asymptomatic infections to severe and potentially fatal ones [2].

At the end of 2013, chikungunya transmission was confirmed in the Americas [3] and in the following year it was reported in Brazil, with more than 300,000 cases being recorded between 2016 and 2017, with approximately 300 deaths [4]. In Ceará, Northeastern Brazil, the first cases were confirmed in October 2015. In the following years, two epidemic waves occurred (2016/2017), with a large number of reported cases (>150,000) and with a record 245 deaths [5,6]. Fortaleza was the municipality that registered the highest number of chikungunya deaths in Brazil, probably due to the large number of cases and a joint action between surveillance, laboratory, death verification service and the arbovirus death investigation committee working together in collaboration [7,8].

Despite this large number of deaths recorded in Brazil, before the outbreak in Réunion, this disease had not been associated with high mortality rates [9]. However, in recent years, some studies have challenged the conventional view of the non-lethal nature of CHIKV [10–13]. There is evidence that advanced age and the presence of comorbidities increase the likelihood of cases progressing more severely [9,14], however the relative importance of each of these and other factors in progression to death is not well known.

The objective of this study was to assess potential factors associated with deaths from CHIK that occurred during the 2017 epidemic in the city of Fortaleza, Brazil.

## Methods

### Study area

**Fig 1:**
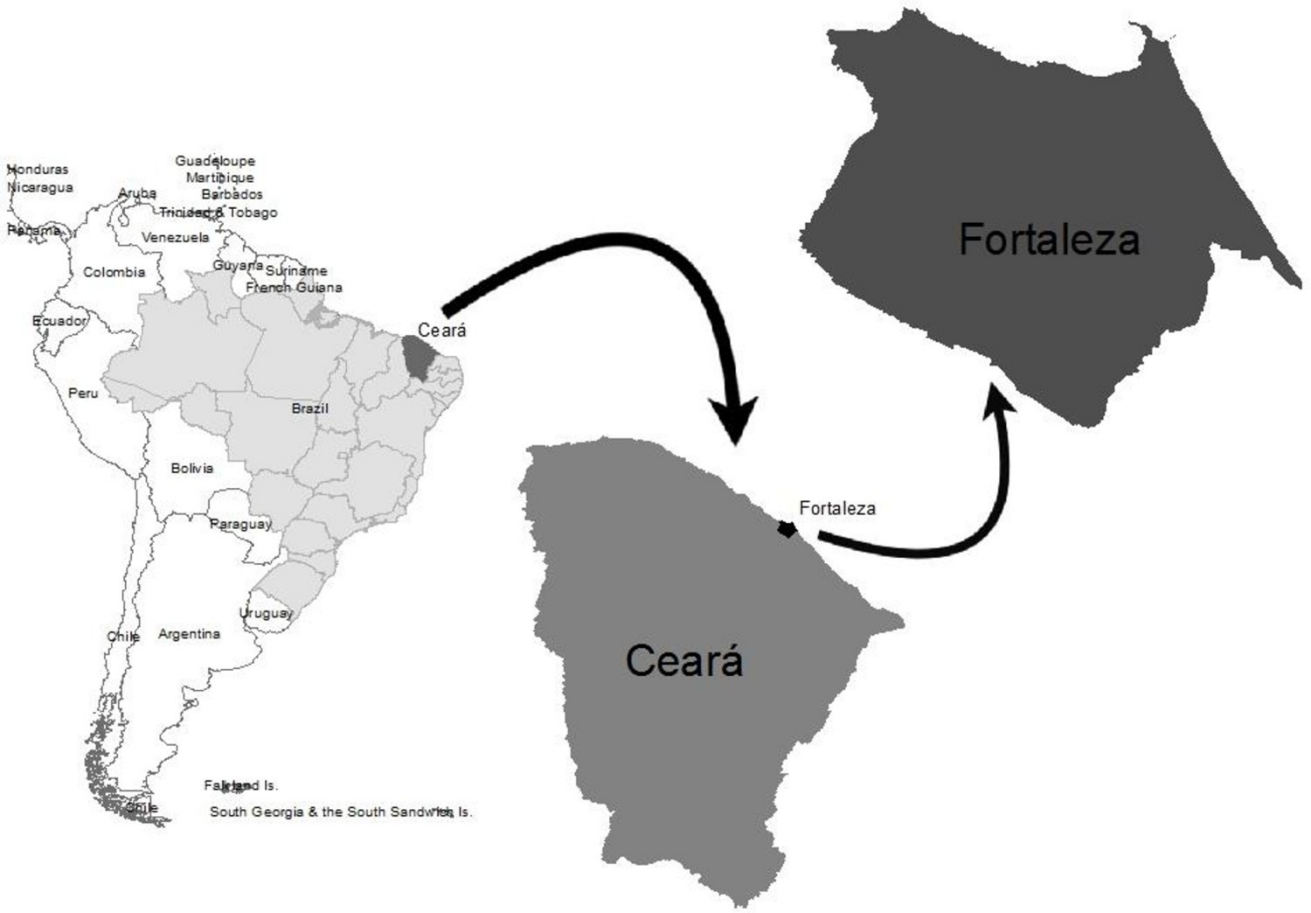
City of Fortaleza, Ceará, Brazil.

This study was carried out in the city of Fortaleza, located in the Northeast region of Brazil (Fig 1). It is considered the 5th most populous state capital in Brazil, with almost 2.5 million inhabitants spread over 315 km^2^. The climatic condition is very favorable for the survival of Aedes mosquitoes, with dengue epidemics registered since 1986 [15].

### Study design

A matched case-control study was conducted (1:2), by sex, age (± 5 years) and neighborhood.

Cases were chikungunya associated deaths between January 1 and December 31, 2017, which were laboratory confirmed by serology (IGM) or by RT-PCR. In the same way, controls were laboratory confirmed CHIK patients (IgM and / or RT-PCR), which occurred in the city of Fortaleza in the same period but did not progress to death. These controls were randomly selected among eligible cases (according to sex and age) reported in the same neighborhood as the deaths, using the Brazilian Ministry of Health disease notification system (SINAN online).

### Source and procedure for data collection

The data were collected by a trained nurse who administered a semi-structured questionnaire during home visits to the controls and to the family members of cases (deaths). The interviews took place between August 2017 and September 2018, after signing an informed consent form. The SINAN database for the city of Fortaleza / CE was used in order to identify the addresses of deaths and confirmed cases.

When necessary, information was complemented by telephone contact and secondary data sources were accessed, such as patient records in the hospital institutions where they were treated, the arbovirus death investigation forms used by the epidemiological surveillance service of the city of Fortaleza, the reports of the Ceará Arbovirus Death Investigation Committee [6], the reports of the Ceará Death Verification Service and the Brazilian Ministry of Health Mortality Information System.

### Data quality control

The procedures for systematic review of the questionnaires and verification of the collected data were carried out in 10% of the sample aiming at the quality and reliability of the collected primary data and correction of consistency and typing errors.

### Variables analyzed

The explanatory variables analyzed were demographic variables, such as sex (male and female), age, race / color (white, multiethnic black, hispanic and black), education (<1 year, 1–3, 4–7, 8–10, 11–14, 15 or more years of study), marital status (single, married, widowed or divorced); medical history (cardiovascular diseases, diabetes, hypertension and others), household environmental characteristics (type of habitation, water storage, etc) as well such as presence of signs and symptoms during the clinical course and also musculoskeletal signs and laboratory tests including amount of platelets and leukocytes such as neutrophils and lymphocytes of those patients who had access to the exams or that were in the medical record (supplementary document). The time between symptom onset and death was categorized into acute (within 20 days), postacute (21–90 days) and late (more than 90 days) [40].

### Data analysis

Data were entered on Epi Info 7.2 software and analyzed using JAMOVI 1.0 and SPSS 20.0. Absolute and relative frequencies were calculated, and the chi-squared and Fisher’s exact tests were used to assess associations between risk factors and death from CHIK. The Mann-Whitney U test was used to analyze the characteristics of the participants. Crude (ORc) and adjusted (ORadj) odds ratios and respective confidence intervals were estimated using multiple logistic regression models. The stepwise forward method was performed to select the final logistic model. Explanatory variables were analyzed by groups of factors, as follows: demographic, behavioral, pre-existing diseases, symptoms, complications and were analyzed within them. After this stage, a final model was run with the variables that were significant in the analysis of each group. A significance level of 5% was adopted. In turn, the variation range explained by the model was described with Nagelkerke R2 values.

### Ethical aspects

The study was approved by the Ethics Committee of the Federal University of Ceará, as per Certificate of Submission for Ethical Appraisal (CAAE) No. 69013017.8.0000.5054.

## Results

**Fig 2:**
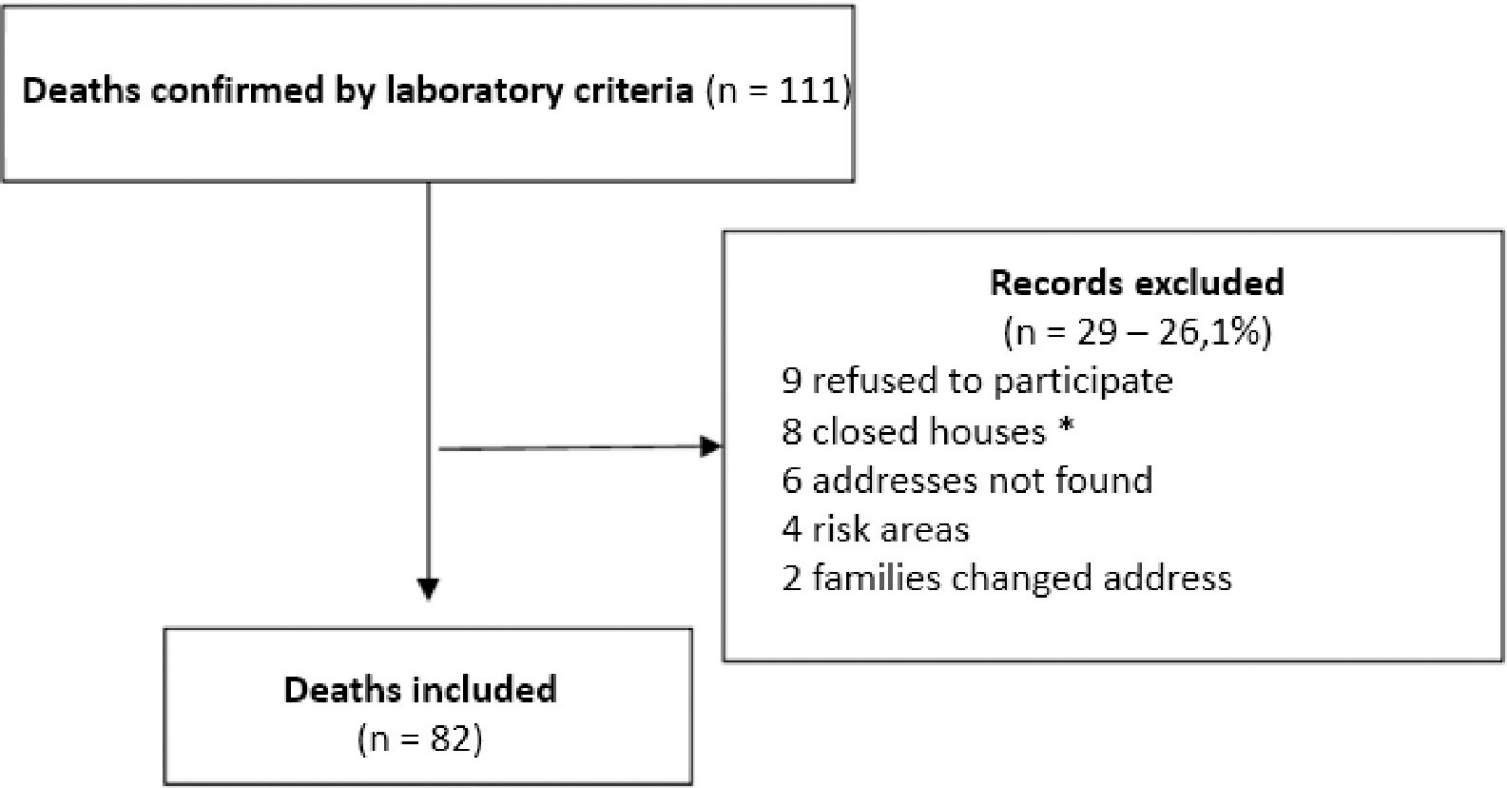
Flowchart of cases investigated during the chikungunya epidemic in the city of Fortaleza in 2017. *After 4 attempts at different times.

In 2017, 111 CHIK deaths were confirmed according to laboratory criteria. Of these, 82 (73.9%) were included in the study and were paired with 164 controls (Fig 2).

Among the deaths, males (56.1%), people of multiethnic black (58.5%), those who had up to 7 years of study (31.6%), were married (50.0%) and sedentary (69.5%) were all predominant (Table 1). Death occurred in the acute phase in 41 (49.4%), in the postacute phase in 38 (45.8%) cases, in the late phase in 2 (2.4%) cases. The time between symptom onset and death was ignored in 1 (1.2%) case.

**Table 1:** Distribution of sociodemographic characteristics of cases (deaths) and controls (survivors). Fortaleza (Brazil), 2017.

Variables	Groups		p-value
	Case N (%)	ControlN (%)	
Sex			
Male	46 (56.1)	92 (56.1)	
Female	36 (43.9)	72 (43.9)	
Age (years)			
0 to 19 ys	2 (1.2)	1 (1.2)	
20 to 39 ys	7 (4.3)	3 (3.6)	
40 to 59 ys	21 (12.8)	8 (9.5)	
60 more ys	134 (81.7)	70 (83.3)	
Race/color			0.010^d^
White	29 (35.4)	88 (53.7)	
Black	5 (6.1)	3 (1.8)	
Hispanic	0 (0.0)	1 (0.6)	
Multiethnic black	48 (58.5)	72 (43.9)	
Years of study			0.441^c^
<1 year	14 (17.1)	19 (11.6)	
1–3 years	13 (15.9)	17 (10.4)	
4–7 years	26 (31.7)	61 (37.2)	
8–10 years	16 (19.5)	36 (22.0)	
11–14 years	6 (7.3)	17 (10.4)	
15 or more	6 (7.3)	14 (8.5)	
No answer	1 (1.2)	0 (0.0)	
Marital status			0.886
Single	13 (15.9)	21 (12.8)	
Married	43 (52.4)	95 (57.9)	
Widowed	19 (23.2)	38 (23.2)	
Divorced	7 (8.5)	10 (6.1)	
Smoking			0.555
Yes in the past	42 (51.2)	73 (44.5)	
Yes and still smoking	4 (4.9)	9 (5.5)	
No	35 (42.7)	82 (50.0)	
No answer	1 (1.2)	0 (0.0)	
Drink alcohol			0.693
Yes	23 (28)	50 (30.5)	
No	59 (72)	114 (69.5)	

Data expressed as mean ± standard deviation and median (25th percentile - 75th percentile) b: Mann-Whitney test; c: Pearson’s chi-square test; d: Fisher’s exact test.

Age at death ranged from 0 to 98 years and median time to death was 19 days. The peak of the onset of symptoms occurred in April 2017 (41.2%).

Considering their clinical history, people with other chronic heart diseases (OR 3.8; 95% CI: 1.53–9.26; p = 0.004) and chronic kidney disease (OR 12.77; 95% CI: 2.75–59.4; p = 0.001) (Nagelkerke R^2^ = 0.819) were identified as having greater likelihood of progressing to death and remained associated after multiple logistic regression. Risk of dying was not associated with any vaccination history.

In the multivariate analysis of the variables related to signs and symptoms, the fever (OR 19.23 CI: 1.73–213.78 p = 0.016), abdominal pain (OR: 3; 74 CI: 1.06–13.16 p = 0.04), apathy (OR: 11.62 CI: 2.95–45.82 p = 0.001) and dyspnea (OR: 50.61; CI: 12.37–207.18; p <0.001) variables were identified with greater likelihood of death from CHIK. On the other hand, symptoms such as pruritus (OR: 0.18; CI: 0.05–0.66; p = 0.01) and retro-orbital pain (OR: 0.15; CI: 0.04–0.55; p <0.005) were significantly more reported by patients who progressed to cure (Table 2).

**Table 2:** Frequency of signs and symptoms of several systems and likelihood of death from Chikungunya. Fortaleza (Brazil), 2017.

Systems	Variables	Groups		Crude analysis		Multiple logistic regression*	
		Case	Control	OR (95%CI)	p-value	OR (95%CI)	p-value
		N (%)	N (%)				
**General symptoms**	Fever	77 (93.9)	120 (73.2)	7.06 (2.44–20.43)	<0.001^c^	19.23 (1.73–213.78)	0.016
	Hypothermia	9 (11.11)	0 (0.0)	45.63 (2.62–79.4)	<0.001^d^		
	Myalgia	51 (62.2)	96 (58.5)	1.29 (0.74–2.25)	0.369^c^		
	Prostration	71 (86.6)	80 (48.8)	9.32 (4.22–20.58)	<0.001^c^		
	Irritability	39 (47.6)	94 (57.3)	0.74 (0.43–1.28)	0.285^c^		
	Apathy	36 (44.0)	32 (19.5)	3.53 (1.96–6.37)	<0.001^c^	11.62 (2.95–45.82)	<0.001
	Postural hypotension	34 (41.5)	24 (14.6)	4.41 (2.37–8.20)	<0.001^c^		
	Lipothymia / fainting	34 (41.5)	47 (28.7)	1.92 (1.10–3.37)	0.021^c^		
**Respiratory symptoms**	Dyspnea	60 (73.2)	16 (9.8)	27.75 (13.47–57.16)	<0.001^c^	50.61 (12.37–207.18)	<0.001
	Cough	31 (37.8)	23 (14.0)	3.88 (2.07–7.28)	<0.001^c^		
	Coryza	8 (9.8)	14 (8.5)	1.22 (0.49–3.05)	0.664^c^		
	Sore throat	7 (8.5)	11 (6.7)	1.40 (0.52–3.77)	0.599^d^		
	Pharyngitis	1 (1.2)	2 (1.2)	1.09 (0.10–12.19)	>0.999^d^		
	Canker sores in mouth and/or throat	25 (30.5)	66 (40.2)	0.75 (0.43–1.35)	0.342^c^		
**Digestive symptoms**	Abdominal pain	40 (48.78)	50 (30.5)	2.44 (1.40–4.27)	0.002^c^	3.74 (1.06–13.16)	0.04
	Diarrhea	41 (50.0)	42 (25.6)	3.13 (1.78–5.51)	<0.001^c^		
	Nausea	41 (50.0)	51 (31.1)	2.39 (1.38–4.15)	0.002^c^		
	Vomiting	32 (39.0)	39 (23.8)	2.14 (1.2–3.79)	0.009^c^		
	Hepatomegaly	5 (6.1)	0 (0.0)	37.14 (2.01–685)	0.001^d^		
**Skin**	Rash	45 (54.88)	74 (45.1)	1.52 (0.89–2.60)	0.124^c^		
	Itching	26 (31.7)	86 (52.4)	0.44 (0.25–0.78)	0.004^c^	0.18 (0.05–0.66)	0.01
	Petechiae	13 (15.9)	38 (23.2)	0.65 (0.33–1.31)	0.229^c^		
	Bruise	6 (7.3)	2 (1.2)	6.75 (1.33–34.25)	0.015^d^		
	Epistaxis	2 (2.4)	0 (0.0)	10.89 (0.51–229)	0.101^d^		
	Hematoma	8 (9.8)	5 (3.1)	3.63 (1.15–11.5)	0.030^d^		
**Central Nervous System**	Headache	52 (63.4)	123 (75.0)	0.55 (0.29–1.01)	0.286^c^		
	Retro-orbital pain	20 (24.4)	74 (45.1)	0.44 (0.24–0.80)	0.007^c^	0.15 (0.04–0.55)	0.005
	Paresis	14 (17.1)	15 (9.2)	2.24 (1.02–4.92)	0.040^c^		
	Paralysis	18 (22.0)	3 (1.8)	16.95 (4.81–59.69)	<0.001^c^		
	Somnolence	73 (89.0)	116 (70.7)	4.32 (1.85–10.05)	<0.001^c^		
**Others**	Dry conjunctivitis	10 (12.2)	24 (14.6)	0.87 (0.39–1.92)	0.732^c^		
	Lymphadenopathy	1 (1.2)	2 (1.2)	1.17 (0.10–13.16)	>0.999^d^		
	Splenomegaly	2 (2.4)	0 (0.0)	15.32 (0.72–324)	0.063^d^		
**Antecedent**	Diabetes	28 (34.1)	37 (22.6)	1.8 (0.9–3.1)	0.052^c^		
	HAS	64 (78.0)	101 (61.6)	2.2 (1.2–4.1)	0.010^c^		
	Heart failure	8 (9.8)	3 (1.8)	5.8 (1.5–22)	<0.001^c^		
	Other chronic heart disease	17 (20.7)	9 (5.5)	4.5(1.9–10.6)	<0.001^c^	3.77 (1.53–9.26)	0.004
	Chronic kidney disease	13 (15.9)	2 (1.2)	15 (3.3–69)	<0.001^d^	12,77 (2,75–59,4)	0.001
	Chronic lung disease	6 (7.3)	16 (9.8)	0.7 (0.2–1.9)	0.527^c^		
	Other chronic disease	41 (50)	86 (52.4)	0.9 (0.5–1.5)	0.718^c^		

Data expressed in n and%; c: Pearson’s chi-square test; d: Fisher’s exact test. * Nagelkerke R^2^ = 0.819.

Among musculoskeletal symptoms, people with arthritis (51.2%) were 7.75 times more likely to die from CHIK (p<0.001). All other symptoms analyzed in this group, such as morning stiffness, joint pain, extent of pain, location, and intensity of joint pain, were not associated with death (Table 3).

**Table 3:** Frequency of musculoskeletal signs and symptoms associated with likelihood of death from Chikungunya. Fortaleza (Brazil), 2017.

Variables	Groups		ORc	p-value
	Case	Control		
	N (%)	N (%)		
Arthritis	42 (51.2)	22 (13.4)	7.75 (4.12–14.61)	**<0.001^c^**
Joint pain	78 (95.12)	161 (98.2)	0.73 (0.12–4.44)	0.664^c^
Joint pain extension				0.351^c^
Oligoarthralgia	19 (24.36)	31 (19.2)	1.36 (0.71–2.60)	
Polyarthralgia	59 (75.64)	131 (80.9)	1	
Pain intensity				0.169^c^
Mild	4 (5.19)	14 (8.6)	1	
Moderate	31 (40.26)	80 (49.4)	1.35 (0.41–4.41)	
Intense	42 (54.55)	68 (42.0)	2.16 (0.67–7.00)	
Pain site				
Head Neck	1 (1.25)	4 (2.4)	0.51 (0.05–4.61)	>0.999^d^
Spine Torso	3 (3.75)	2 (1.2)	3.16 (0.52–19.28)	0.334^d^
Sacral spine	3 (3.75)	9 (5.5)	0.67 (0.17–2.55)	0.756^d^
Shoulder	7 (8.75)	12 (7.3)	1.21 (0.46–3.21)	0.695^c^
Elbow	4 (5)	9 (5.5)	0.91 (0.27–3.04)	>0.999^d^
Fist	6 (7.5)	22 (13.4)	0.52 (0.20–1.35)	0.204^c^
Fingers	4 (5)	17 (10.4)	0.45 (0.15–1.40)	0.161^c^
Toes	1 (1.25)	7 (4.3)	0.28 (0.3–2.35)	0.279^d^
Soles of the feet	10 (12.5)	28 (17.1)	0.69 (0.32–1.51)	0.355^c^
Hip	2 (2.5)	5 (3.1)	0.82 (0.15–4.30)	>0.999^d^
Knees	24 (30)	35 (21.3)	1.58 (0.86–2.90)	0.138^c^
Ankle	13 (16.25)	32 (19.5)	0.80 (0.39–1.63)	0.537^c^
All joints	36 (45)	83 (50.9)	0.79 (0.46–1.35)	0.386^c^
Morning stiffness	67 (81.71)	150 (91.5)	0.58 (0.24–1.39)	0.218^c^

Data expressed in n and%; c: Pearson’s chi-square test; d: Fisher’s exact test.

Among the laboratory findings analyzed, only two were not associated with greater likelihood of death: lymphocytes and albumin (p>0.05). Greater likelihood of progressing to death was observed among patients who presented thrombocytopenia (OR: 10.1; CI: 3.9–26.3; p <0.001), leukopenia (OR: 7.4; CI: 2.4–23.0; p <0.001) and leukocytosis (OR = 14; IC = 5.4–36.5; p <0.001). Neutropenia and lymphopenia were also associated with greater likelihood of death with odds ratios (OR) of 6.4 and 14.2, respectively (p = 0.001). Other laboratory markers also showed an association with evolution to death, such as CRP> 3 (OR: 12.4; CI: 2.9–52.6; p <0.001), TGO> 40 (OR: 9.4; CI: 3, 1–28.6; p <0.001), TGP> 45 (OR: 3.2; CI: 1.1–9.1; p = 0.027), urea> 45 (OR: 17.8; CI: 5, 5–57.3; p <0.001) and creatinine <1.3 (OR: 17.8; CI: 5.8–54.2; p <0.001). It also stood out that altered blood glucose was associated with cases with a worse prognosis (OR: 13.5; CI: 1.3–135.0; p = 0.038) (Table 4).

**Table 4:** Laboratory tests of cases (deaths) and controls (survivors).

Laboratory tests	Groups		ORc (95%CI)	p
	Case N (%)	Control N (%)		
Platelets (n = 113)				**<0.001**
<150,000 mm^3^	51 (68.92)	7 (17.95)	10.1 (3.9–26.3)	
>150,000 mm^3^	23 (31.08)	32 (82.05)	1	
Leukocytes_min (n = 113)				**<0.001**
<3500 mm^3^	34 (45.95)	4 (10.26)	7.4 (2.4–23)	
>3500 mm^3^	40 (54.05)	35 (89.74)	1	
Leukocytes_max (n = 113)				**<0.001**
>10000 mm^3^	58 (78.4)	8 (100.0)	14.1(5.4–36.5)	
<10000 mm^3^	16 (21.6)	31 (0.0)	1	
Neutrophils _min (n = 82)				**<0.001**
<1500 mm^3^	28 (60.87)	7 (19.44)	6.4 (2.3–17.8)	
>1500 mm^3^	18 (39.13)	29 (80.56)	1	
Neutrophils_max (n = 82)				**0.008**
>7500 mm^3^	13 (28.26)	2 (5.56)	6.7 (1.4–32)	
<7500 mm^3^	33 (71.74)	34 (94.44)	1	
Lymphocytes _min (n = 99)				**<0.001**
<1000 mm^3^	52 (85.25)	11 (28.95)	14.2 (5.2–38.4)	
>1000 mm^3^	9 (14.75)	27 (71.05)	1	
Lymphocytes _max (n = 99)				>0.999
>3500 mm^3^	3 (4.92)	2 (5.26)	0.9 (0.1–5.8)	
<3500 mm^3^	58 (95.08)	36 (94.74)	1	
CRP_max (n = 48)				**<0.001**
>3 mg/dL	27 (81.82)	4 (26.67)	12.4 (2.9–52.6)	
<3 mg/dL	6 (18.18)	11 (73.33)	1	
TGO_max (n = 74)				**<0.001**
>40 U/L	40 (81.63)	8 (32.0)	9.4 (3.1–28.6)	
<40 U/L	9 (18.37)	17 (68.0)	1	
TGP_max (n = 72)				**0.027**
>45 U/L	26 (55.32)	7 (28.0)	3.2 (1.1–9.1)	
<45 U/L	21 (44.68)	18 (72.0)	1	
Urea_max (n = 91)				**<0.001**
>45 mg/dl	60 (90.91)	9 (36.0)	17.8 (5.5–57.3)	
<45 mg/dl	6 (9.09)	16 (64.0)	1	
Creatinine_max (n = 94)				**<0.001**
<1.3 mg/dL	59 (89.39)	9 (32.14)	17.8 (5.8–54.2)	
>1.3 mg/dL	7 (10.61)	19 (67.86)	1	
Albumin_max (n = 21)				0.128
<3.5 g/L	3 (16.67)	2 (66.67)	0.1 (0–1.5)	
>3.5 g/L	15 (83.33)	1 (33.33)	1	
Glucose_max (n = 25)				**0.038**
>125 mg/dL	3 (60.0)	2 (10.0)	13.5 (1.3–135)	
<125 mg/dL	2 (40.0)	18 (90.0)		

Data expressed in n and %; c: Pearson’s chi-square test; d: Fisher’s exact test. min = lowest values; max = highest values.

## Discussion

This study identified a predominance of males, people of multiethnic black, with up to 7 years formal education, married and over 65 years of age. In addition, we found that the factors most strongly associated with the deaths of people with CHIK were: chronic kidney disease and previous heart disease, presence of fever, abdominal pain, apathy, dyspnea and arthritis, as well as laboratory findings such as leukocytosis, leukopenia, thrombocytopenia, neutropenia and lymphopenia.

Having a record of fever increased likelihood of death 16 times. This symptom has been reported as being more frequent among people who died in other cities around the world [16]. In addition, having a record of high fever has also been associated with patients with severe chikungunya who required hospitalization [9,17,18]. This symptom is likely to be very intense due to the increase in pro-inflammatory cytokines such as IL-6 or IFN-α which are stimulated as a result of viral infection. Economopoulou et al. [9] revealed that high fever increased substantially in patients who used non-steroidal anti-inflammatory drugs (NSAIDs) before hospitalization which can lead to a more severe form of the disease or later hospitalization. However, the role of specific NSAIDs (aspirin or paracetamol) in the disease has not yet been clearly determined and needs to be investigated. It should be noted that fever with the presence of arthralgia is one of the most sensitive markers with regard to the identification of suspected chikungunya cases [17].

Another symptom that proved to be a manifestation related to increased mortality from chikungunya was dyspnea. This finding also appears in the study by Alvarez et al. [19] characterizing both a symptom of cardiovascular failure aggravated by arbovirus and a symptom of respiratory dysfunction which may be due to a more severe condition of the disease which involves the cardiovascular and respiratory system [12]. This fact was also evidenced in patients with arboviruses who progressed to worse outcomes [20]. As previously mentioned, cardiovascular diseases are also a predictive factor for a worse outcome and they alone cause respiratory manifestations such as difficulty in breathing. In addition, it is known that CHIKV infection tends to exacerbate other pre-existing diseases generating decompensation [21].

Furthermore, in our study severe abdominal pain was associated with greater likelihood of death from chikungunya. This result was also found in studies by Bonifay et al. [22]. The authors defined it as an atypical and severe manifestation of the disease due to hepatitis and acute pancreatitis with a worse prognosis in patients with a history of alcoholism and older than 85 years [22].

Apathy was also significantly associated with a greater likelihood of progressing to death. Work by Cunha and Trinta [23] in Brazil pointed out that 4.6% of patients declared themselves extremely depressed and 35.5% felt unmotivated to perform daily activities, revealing great apathy. This is probably due to persistent arthralgia that usually affects multiple joints causing functional loss, reduced quality of life and symptoms of asthenia, depression, and anxiety [24].

This study detected significant association of chronic kidney disease as a factor that increased the likelihood of death in chikungunya, probably due to a central component of kidney injury. This same finding was observed in other studies, such as that by Brito [25], in which some deaths occur indirectly due to infection mainly resulting from decompensation of previous comorbidities, which include patients with kidney, heart or lung diseases [26]. Comorbidities such as hypertension and diabetes are associated with greater chronicity as well as with worse outcomes or with increased pain severity [17]. It is known that hypertension and diabetes are predictive factors for the progression of kidney disease and that there is decompensation of underlying diseases in patients with CHIKV infection [21,27]. This can be a contributing factor to severe evolution in these patients [17], especially when these comorbidities are decompensated [28].

With regard to pre-existing cardiovascular diseases, heart failure, systemic arterial hypertension (SAH) and chronic heart disease were recognized in the study as factors associated with death from chikungunya. This observation is in line with what has been documented by Alvarez et al. [19] who identified that patients with cardiovascular comorbidities showed faster deterioration and worse prognosis. It should be added that as recorded in the work of Azevedo, Oliveira & Vasconcelos [29], chikungunya can still present cardiac alterations, especially myocarditis, pericarditis, and dilated cardiomyopathy. One of the explanations for this cardiovascular impairment is the possible cardiac tropism of CHIKV, which upon penetrating myocytes generates direct damage to muscle fibers, culminating in an inflammatory response which results in damage to cardiac tissue and necrosis [19,30].

In this study, itching and retro-orbital pain were considered protective factors against death from chikungunya. Retro-orbital pain is a symptom reported in other cases [31]. This finding is justified because both symptoms are self-limiting occurring in the acute phase of the disease and lasting approximately 1 week when IgM anti-CHIKV antibodies appear [32]. On the other hand, these are symptoms very often reported in patients with dengue or Zika [33] and may be associated with false-positive cases. In addition, according to the study by Caglioti et al. [34], most eye manifestations have a benign course with complete resolution and preservation of vision.

We demonstrated that prior presence of arthritis significantly increased the likelihood of progressing to death. Despite the persistence of musculoskeletal complaints being the main characteristic of chikungunya, there are some factors that are associated with a worse prognosis. Some studies show that prominent joint involvement in the acute phase (joint edema and stiffness, polyarthritis, tenosynovitis) can be a marker for worse disease progression [35]. Therefore, prior presence of arthritis may have contributed to patients having worse outcomes. This may be due to direct tissue damage induced by the virus; long-term persistence of CHIKV infection in tissues with concomitant inflammation; and activation of autoimmune responses [36].

We found a set of laboratory markers that can be important markers of poor prognosis and that are found in blood counts, this being an examination that is part of the routine investigation of any infectious condition. Among the markers of poor prognosis are leukocytosis and leucopenia, as well as thrombocytopenia, neutropenia, and lymphopenia. These laboratory findings have already been described as severity markers in patients with dengue fever, another arbovirus disease very common in Brazil [37]. It is well established that laboratory abnormalities are observed in CHIKV infection with a direct correlation with higher viral load [38]. It is important to highlight that patients with thrombocytopenia are often patients with worse prognosis both in the pediatric population and in adults [16,39,40]. Leukopenia has also been associated with greater severity and need for hospitalization [18]. Changes in liver enzymes, increased urea creatinine and elevated CRP were also good predictors of death and should be considered in the presence of a patient with clinical suspicion of chikungunya. There is also evidence that patients with CHIK may go on to suffer albuminuria, hematuria, nephritis, kidney damage and other kidney function abnormalities [19,28]. We report here that renal markers such as creatinine and urea showed significant alteration.

The main limitation of this study is due to the possible memory bias of the cases that survived and even more so among the relatives of the people who died. This bias has been minimized, as far as possible, by searching for information in medical records, surveillance records, laboratories and hospitals attended by each case and control. Another aspect that deserves to be highlighted is that even though all these deaths have undergone exhaustive investigation by the expert committee [6], it is always difficult to define precisely whether disease manifestation was serious due to the presence or exacerbation of existing comorbidities or, alternatively, whether comorbidities decompensate due to severe CHIK, which would suggest the potential for reverse causality bias. Additionally, it is difficult to establish whether people died with chikungunya or from chikungunya [7,13,40].

Many more prospective studies will be necessary for us to fully understand chikungunya mortality. However, in this study we found that the factors most strongly associated with death, such as chronic kidney disease and previous heart disease and some signs and symptoms, such as fever, abdominal pain, apathy, dyspnea and arthritis, should serve as a warning to professionals for closer monitoring of this type of patient. In addition, blood count findings such as leukocytosis, leucopenia, thrombocytopenia, neutropenia and lymphopenia should draw attention to the risk of severity and reinforce the importance of complete blood tests as part of investigation of all patients with suspected chikungunya.

## Supporting information

S1 File (DOC)

S2 File (DOC)

## References

[1] Weaver SC. Arrival of Chikungunya virus in the new world: prospects for spread and impact on public health. PLoS Negl Trop Dis. 8(6):e2921. 2014. doi: 10.1371/journal.pntd.0002921 24967777 PMC4072586

[2] Silva LA, Dermody TS. Chikungunya virus: epidemiology. replication. disease mechanisms. and prospective intervention strategies. J Clin Invest. v. 127. p. 737–49. 2017. doi: 10.1172/JCI84417 28248203 PMC5330729

[3] Burt FJ, Rolph MS, Rulli NE, et al. Chikungunya: a re-emerging virus. *Lancet Infect Dis*. 2012. 379(9816):662–71. doi: 10.1016/S0140-6736(11)60281-X 22100854

[4] Brasil. Ministério da Saúde. Secretaria de Vigilância em Saúde. Secretaria de Atenção Básica Chikungunya: Manejo Clínico/ Ministério da Saúde. Secretaria de Vigilância em Saúde. Secretaria de Atenção Básica.–Brasília: Ministério da Saúde. 2017.

[5] Simião AR, Barreto FKA, Oliveira RMAB, et al. A major chikungunya epidemic with high mortality in northeastern Brazil. *Rev*. *Soc*. *Bras*. *Med*. *Trop*. [online]. 2019. vol.52. e20190266. Epub Oct 03. 2019. ISSN 1678-9849. doi: 10.1590/0037-8682-0266-2019 31596354

[6] Cavalcanti LPG, Escóssia KNF, Simião AR, et al. Experience of the Arbovirus Death Investigation Committee in Ceará. Brazil. in 2017: advances and challenges. Epidemiol. Serv. Saude. Brasília. 28(3):e2018397. 2019. doi: 10.5123/S1679-49742019000300011 31800868

[7] Cavalcanti LPG, Freitas ARR, Brasil P, et al. Surveillance of deaths caused by arboviruses in Brazil: from dengue to chikungunya. Mem Inst Oswaldo Cruz. 2017. Rio de Janeiro. Vol. 112(8): 583–585. doi: 10.1590/0074-02760160537 28767985 PMC5530552

[8] Cavalcanti LPG, Braga DNM, Silva LMA, et al. Postmortem Diagnosis of Dengue as an Epidemiological Surveillance Tool. Am. J. Trop. Med. Hyg. 94(1). 2016. pp. 187–192 doi: 10.4269/ajtmh.15-0392 26598561 PMC4710428

[9] Economopoulou A, Dominguez M, Helynck B, et al. Atypical Chikungunya virus infections: clinical manifestations. mortality and risk factors for severe disease during the 2005–2006 outbreak on réunion. Epidemiol infect. 2009;137:534–41. doi: 10.1017/S0950268808001167 18694529

[10] de la Hoz JM, Bayona B, Viloria S, et al. Fatal cases of Chikungunya virus infection in Colombia: diagnostic and treatment challenges. J Clin Virol. 2015; 69(8): 27–9. 26209372 10.1016/j.jcv.2015.05.021

[11] Freitas ARR, Alarcón-Elbal PM, Donalisio MR. Excess mortality in Guadeloupe and Martinique. islands of the French West Indies. during the chikungunya epidemic of 2014. Epidemiology and Infection146. 2018. doi: 10.1017/S0950268818002315 30152293 PMC6453005

[12] Freitas ARR, Donalisio MR, Alarcón-Elbal PM. Excesso de mortalidade e causas associadas à chikungunya. Porto Rico. 2014–2015. *Emerg Infect Dis*. 2018;24(12):2352–2355. doi: 10.3201/eid2412.170639 30277456 PMC6256393

[13] Frutuoso LCV, Freitas ARR, Cavalcanti LPG, et al. Estimated mortality rate and leading causes of death among individuals with chikungunya in 2016 and 2017 in Brazil. *Journal of the Brazilian Society of Tropical Medicine*. Vol.:53:e20190580: 2020. doi: 10.1590/0037-8682-0580-2019 32294696 PMC7182291

[14] Crosby L, Perreau C, Madeux B, et al. Severe manifestations of chikungunya virus in critically ill patients during the 2013–2014 Caribbean outbreak. Int J Infect Dis. 2016;48:78–80. doi: 10.1016/j.ijid.2016.05.010 27208636

[15] Cavalcanti LPG, Barreto FKA, Oliveira RMAB, et al. Thirty years of dengue in Ceará: history. contributions to science and challengesin the current scenario with triple arbovirus circulation.J. Health Biol Sci. 2018; 6(1):65–82.

[16] Mercado M, Acosta-Reyes J, Parra E, et al. Clinical and histopathological features of fatal cases with dengue and chikungunya virus co-infection in Colombia. 2014 to 2015. Euro Surveill. 2016; 21(22):pii = 30244. doi: 10.2807/1560-7917.ES.2016.21.22.30244 27277216

[17] Badawi A, Ryoo SG, Vasileva D, et al. Prevalence of chronic comorbidities in chikungunya: A systematic review and meta-analysis. *Int J Infect Dis*. 2018;67:107–113. doi: 10.1016/j.ijid.2017.12.018 29277382 PMC7110669

[18] Pinto JR, Silva JGB, Mota RMS, et al. Clinical profile and factors associated with hospitalization during a Chikungunya epidemic in Ceará. Brazil. Rev. Soc. Bras. Med. Trop. [Internet]. 2019 [cited 2020 July 18]; 52:e20190167. Available from: http://www.scielo.br/scielo.php?script=sci_arttext&pid=S0037-86822019000100688&lng=en. Epub Oct 03. 2019. doi: 10.1590/0037-8682-0167-2019 31596350

[19] Alvarez MF, Bolívar-Mejía A, Rodriguez-Morales AJ, et al. Cardiovascular involvement and manifestations of systemic Chikungunya virus infection: a systematic review. F1000research. Risaralda. v. 6. p. 390. 2 maio 2017. F1000 Research Ltd. doi: 10.12688/f1000research.11078.2 28503297 PMC5405794

[20] Gupta N, Mittal A, Kutty SV, et al. Technical and Alarm signs for referral in adult patients with acute febrile illness: A study from a tertiary care hospital in North India. J Family Med Prim Care. 2018;7(4):832–835. doi: 10.4103/jfmpc.jfmpc_138_18 30234063 PMC6132017

[21] Barreto FKA, Montenegro Jr. RM, Fernandes VO, et al. Chikungunya and diabetes. what do we know?. Diabetology & Metabolic Syndrome. 2018. v. 10. n. 32. p. 1–6. 29686737 10.1186/s13098-018-0329-2 PMC5899414

[22] Bonifay T, Prince C, Neyra C, et al. Atypical and severe manifestations of chikungunya virus infection in French Guiana: a hospital-based study. Plos One. [s.l.]. v. 13. n. 12. p. 1–13. 6 dez. 2018. Public Library of Science (PLoS). 10.1371/journal.pone.0207406 PMC6283639 30521555

[23] Cunha RV, Trinta KS. Chikungunya virus: clinical aspects and treatment—a review. Memórias do Instituto Oswaldo Cruz. [s.l.]. v. 112. n. 8. p. 523–531. ago. 2017. FapUNIFESP (SciELO). doi: 10.1590/0074-02760170044 28767976 PMC5530543

[24] Sam I, Kümmerer BM, Chan YF, et al. Updates on Chikungunya Epidemiology. Clinical Disease. and Diagnostics. Vector-borne And Zoonotic Diseases. [s.l.]. v. 15. n. 4. p. 223–230. abr. 2015. Mary Ann Liebert Inc. doi: 10.1089/vbz.2014.1680 25897809

[25] Brito CAA. Alert: Severe cases and deaths associated with Chikungunya in Brazil. Revista da Sociedade Brasileira de Medicina Tropical. 50. n. 5. p.585–589. out. 2017. doi: 10.1590/0037-8682-0479-2016 29160503

[26] Mercado M, Acosta-Reyes J, Parra E, et al. Renal involvement in fatal cases of chikungunya virus infection. Journal of Clinical Virology. 2018. n. 103. p. 16–18. doi: 10.1016/j.jcv.2018.03.009 29604514

[27] Cavalcanti LPG, D’angelo SM, Lemos DRQ, et al. Is the recent increment in attributable deaths to type-2 diabetes (T2D) associated with the latest chikungunya outbreak in a major epidemic area in Brazil? Rev Bras Med Trop. 2018. v. 51. p:63–65. 10.1590/0037-8682-0440-2017 29513844

[28] Silva Jr GBD, Pinto JR, Mota RMS, et al. Risk factors for death among patients with Chikungunya virus infection during the outbreak in northeast Brazil. 2016–2017. Trans R Soc Trop Med Hyg. 2018. Dec 14. doi: 10.1093/trstmh/try127 30551206

[29] Azevedo RSS, Oliveira CS, Vasconcelos PFC. Chikungunya risk for Brazil. Rev saúde pública. 2015. V. 49. N.58. 10.1590/S0034-8910.2015049006219 PMC4617438 26398876

[30] Simon F, Paule P, Oliver M. Chikungunya virus-induced myopericarditis: toward an increase of dilated cardiomyopathy in countries with epidemics? Am J Trop Med Hyg. 2008. v.78. n. 2. p. 212–3. 18256416

[31] Vairo F, Mammone A, Lanini S, et al. Local transmission of chikungunya in Rome and the Lazio region. Italy. PLoS ONE 13. 2018 (12): e 0208896. doi: 10.1371/journal.pone.0208896 30576334 PMC6303016

[32] Vairo F, Haider N, Kock R, et al. Chikungunya epidemiology. pathogenesis. clinical features. management. and prevention. Infect Dis Clin N Am 33 (2019) 1003–1025 10.1016/j.idc.2019.08.006id.theclinics.com0891-5520/19/a2019. 31668189

[33] Azeredo EL, Dos Santos FB, Barbosa LS, et al. Clinical and Laboratory Profile of Zika and Dengue Infected Patients: Lessons Learned From the Co-circulation of Dengue. Zika and Chikungunya in Brazil. *PLoS Curr*. 2018; ecurrents. outbreaks. 0bf6aeb4d30824de63c4d5d745b217f5. Published 2018 Feb 15. doi: 10.1371/currents.outbreaks0bf6aeb4d30824de63c4d5d745b217f5 PMC5843488 29588874

[34] Cagliote C. Chikungunya virus infection: an overview. New Microbiologica. Roma, p. 211–227. 30 maio 2013. 23912863

[35] Marques CDL, Duarte ALBP, Ranzolin A, et al. Recomendações da sociedade brasileira de reumatologia para diagnóstico e tratamento da febre chikungunya. Parte 1—Diagnóstico e situações especiais. Revista Brasileira de reumatologia. 2017.

[36] McCarthy MK, Morrinson TE. Chronic chikungunya virus musculoskeletal disease: what are the underlying mechanisms? Future Microbiol. (2016) 11(3). 331–334. doi: 10.2217/fmb.15.147 26939523 PMC4842256

[37] Kularatnam GAM, Jasinge E, Gunasena S, et al. Evaluation of biochemical and haematological changes in dengue fever and dengue hemorrhagic fever in Sri Lankan children: a prospective follow up study. BMC Pediatr. 2019 Apr 1;19(1):87. doi: 10.1186/s12887-019-1451-5 30935373 PMC6442420

[38] Chow A, Her Z, Ong EKS, et al. Persistent arthralgia induced by Chikungunya virus infection is associated with interleukin-6 and granulocyte macrophage colony-stimulating factor. J Infect Dis. 2011. v. 203. n. 2. p:149–57. doi: 10.1093/infdis/jiq042 21288813 PMC3071069

[39] Oliveira RMAB, Barreto FKA, Maia AMPC, et al. Maternal and infant death after probable vertical transmission of chikungunya virus in Brazil–case report. BMC Infect Dis. 2018. v. 18. n. 1. p. 333. doi: 10.1186/s12879-018-3243-1 30012112 PMC6048842

[40] Lima STS, Souza WM, Cavalcante JW, et al. Fatal outcome of chikungunya virus infection in Brazil. Clin Infect Dis. 2021 Oct 5;73(7):e2436–e2443 doi: 10.1093/cid/ciaa1038 32766829 PMC8492446

