# Supplementary material for: Chikungunya Death Risk Factors in Brazil, in 2017: A case-control study
Source: PLoS One. 2022 Apr 7;17(4):e0260939. doi: 10.1371/journal.pone.0260939 (PMC8989201; doi:10.1371/journal.pone.0260939)
Supplement: S1 File — (DOC) [file pone.0260939.s001.doc]

FATORES ASSOCIADOS AOS ÓBITOS POR CHIKUNGUNYA: UM ESTUDO DE CASO CONTROLE na cidade de fortaleza, ceará

**Questionário N º________**

**CASO [ ] CONTROLE [ ]**

**Pesquisador__________________________________ Data da coleta: _____/_____/______**

**Entrevistado: _______________________________________________________________**

**Parentesco do caso/controle: __________________________________________________**

**Telefone(s) de contato:________________________________________________________**

**Endereço do contato:_________________________________________________________**

**1. Identificação e dados demográficos**

1.1 NOME:_________________________________________________________________________

1.2 DATA DE NASCIMENTO: ____/____/_____ 1.3 IDADE:_____anos 1.3.1 IDADE:_____meses

1.4 SEXO: [ ] Masculino [ ] Feminino 1.5 PESO:_______Kg 1.6 ESTATURA:_____cm

1.7 RAÇA/COR: [ ] Branca [ ] Preta [ ] Amarela [ ] Parda [ ] Ignorado

1.8 ESCOLARIDADE: [ ] nenhum ou menos de 1 ano [ ] 1 a 3 anos [ ] 4 a 7 anos [ ] 8 a 10 anos [ ] 11 a 14 anos [ ] 15 anos ou mais [ ] Não respondeu.

1.9 ESTADO CIVIL: [ ] Solteiro(a) [ ] Casado(a) [ ] Viúvo(a) [ ] Separado(a)/Divorciado(a) [ ] União estável [ ] Ignorado

1.10 TINHA/TEM ALGUMA RELIGIÃO? [ ] Sim [ ] Não

Se sim: Qual?_______________________________________________________________________

1.11 OCUPAÇÃO:__________________________________________________________________

1.12 QUANTAS PESSOAS MORAM NA CASA?_________(número)

1.13 QUAL É A RENDA FAMILIAR MENSAL: _________________ REAIS (aproximada)

1.14 ENDEREÇO ATUAL

ESTADO: ________ MUNICÍPIO:_____________________________________________________ RUA/AVENIDA:__________________________________________NÚMERO:________________ BAIRRO: ____________________________________ FONE: ______________________________

1.15 MOROU EM OUTRO ENDEREÇO ANTES/DURANTE O ADOECIMENTO?

[ ] Sim [ ] Não

- Se sim:

ESTADO: _____ MUNICÍPIO:________________________________________________________

RUA/AVENIDA:____________________________________________________________________ NÚMERO:_______BAIRRO:__________________________________________________________

1.16 LOCAL DE TRABALHO OU ATIVIDADE?_________________________________________

1.17 LOCAL PROVÁVEL DE INFECÇÃO?______________________________________________

1.18 MAIS ALGUÉM TEVE CHIKUNGUNYA EM CASA? [ ] Sim [ ] Não [ ] Não sabe

1.19 MAIS ALGUÉM TEVE CHIKUNGUNYA NA RUA? [ ] Sim [ ] Não [ ] Não sabe

**2. Antecedentes**

2.1 ALGUMA VEZ APRESENTOU ALTERAÇÃO DO NÍVEL DE GLICOSE?

[ ] Sim [ ] Não [ ] Não sabe

2.2 TINHA OU TEM PRESSÃO ALTA?

[ ] Sim [ ] Não [ ] Não sabe

2.3 TEM PARENTES DE PRIMEIRO GRAU (pais ou irmãos ou filhos) com diabetes?

[ ] Sim [ ] Não [ ] Não sabe – Se sim: QUAL?___________________________________________

2.4 TEM PARENTES DE SEGUNDO GRAU (tios, tias, avós e primos em primeiro grau) com diabetes? [ ] Sim [ ] Não [ ] Não sabe – Se sim: QUAL?___________________________________

2.5 ALGUMA VEZ UM MÉDICO OU OUTRO PROFISSIONAL DE SAÚDE DISSE QUE TEM/TEVE ALGUMA DESSAS DOENÇAS?

[ ] Sim [ ] Não [ ] Não sabe - Se sim, qual (is):

[ ] Diabetes [ ] Colesterol alto [ ] Angina [ ] Infarto[ ] Derrame [ ] Insuficiência Cardíaca [ ] Hipertensão arterial sistêmica [ ] Cardiopatia crônica [ ] Depressão [ ] Ansiedade [ ] Artrite [ ] Artrose [ ] Febre reumática [ ] Doença renal crônica [ ] Pneumopatias crônicas [ ] Asma [ ] Câncer [ ] Lúpus [ ] Doença neuroléptica [ ] Anemia falciforme [ ] Talassemia [ ] Dengue [ ] Zika [ ] Mayaro [ ] Malária [ ] Leptospirose

[ ]alergias, especificar___________________________________________________

[ ] Outras doenças reumatológicas _________________________________________

[ ] Outra doença autoimune _______________________________________________

[ ]Outras, especificar____________________________________________________

2.6 FAZIA USO DE ALGUM MEDICAMENTO CONTÍNUO?

[ ] Sim [ ] Não [ ] Não sabe - Se sim:

| **Classe** | **Especificar o medicamento e dose** | **Data de início** | **Data de término** |
| --- | --- | --- | --- |
| Corticóides |  | ____\____\_____ | ____\____\_____ |
| Antiinflamatório |  | ____\____\_____ | ____\____\_____ |
| Antibióticos |  | ____\____\_____ | ____\____\_____ |
| Antivirais |  | ____\____\_____ | ____\____\_____ |
| Anticoagulantes |  | ____\____\_____ | ____\____\_____ |
| Anticonvulsivantes |  | ____\____\_____ | ____\____\_____ |
| Ansiolítico |  | ____\____\_____ | ____\____\_____ |
| Anti-hipertensivo |  | ____\____\_____ | ____\____\_____ |
| Diabetogênicos |  | ____\____\_____ | ____\____\_____ |
| Insulina |  | ____\____\_____ | ____\____\_____ |
| Outros |  | ____\____\_____ | ____\____\_____ |

2.7 HOUVE DESCOMPENSAÇÃO CLÍNICA DA ENFERMIDADE CRÔNICA (por exemplo: necessidade de aumentar dosagem medicamentosa)? [ ] Sim [ ] Não [ ] Não sabe - Se sim, especificar: ____________________________________________________________________________________________________________________________________________________________________________________________________________________________________________________________________________________________________________________________________________________________________________________________________________________________________________________________________________________________________________

**3. Hábitos de vida**

**3.1** JÁ FUMOU CIGARROS?

[ ] Sim, no passado, mas não atualmente. Quanto tempo faz que parou?_________________________

[ ] Sim, ainda fumava/fumo. Em média quantos por dia?____________________________________

[ ] Não [ ] Não sabe

**3.2** DURANTE OS ÚLTIMOS 12 MESES COM QUE FREQUÊNCIA MÉDIA O CASO/CONTROLE TEM INGERIDO BEBIDA ALCOÓLICA?

[ ] Bebe/bebia diariamente [ ] Bebe/bebia 1 a 3 vezes por semana [ ] Bebe/bebia 4 a 6 vezes por semana [ ] Bebe/bebia 1 a 3 vezes por mês [ ] menos de 1 vez por mês [ ] Nenhuma [ ] Não sabe

– se sim, quantas doses ou drinks ou cervejas costumava/costuma tomar?_______________________

**3.3 ATIVIDADE FÍSICA**

**3.1** PRATICAVA/PRATICA ALGUMA ATIVIDADE FÍSICA?

[ ] Sim [ ] Não [ ] Não sabe – se sim, quantos dias da semana?______________________________

Quanto tempo em média durava/dura a atividade física?___________________minutos

**4. Histórico vacinal**

| **Imunobiológico** | **Tomou vacina?** | **Nº Doses** | **Data 1º Dose** | **Data2º Dose** | **Data 3º Dose** |
| --- | --- | --- | --- | --- | --- |
| Influenza | [ ] Sim [ ] Não  [ ] Não sabe |  |  |  |  |
| dT | [ ] Sim [ ] Não  [ ] Não sabe |  |  |  |  |
| Dtpa | [ ] Sim [ ] Não  [ ] Não sabe |  |  |  |  |
| Hepatite B | [ ] Sim [ ] Não  [ ] Não sabe |  |  |  |  |
| Tríplice viral | [ ] Sim [ ] Não  [ ] Não sabe |  |  |  |  |
| Febre Amarela | [ ] Sim [ ] Não  [ ] Não sabe |  |  |  |  |
| Dengue | [ ] Sim [ ] Não  [ ] Não sabe |  |  |  |  |

**5. Histórico da doença e de atendimento**

4.1 DATA DE INICIO DOS SINTOMAS:____/____/_______

4.2 Iniciou com que: [ ] febre [ ] dores articulares [ ] exantema [ ] dor nas costas [ ] dor em outras partes do corpo [ ] outros_____________________________________________________________

4.3 SINAIS E SINTOMAS: [1] Sim [2] Não [3] Não Informado

| [ ] Febre Data início: ___/___/____ Duração (dias):____ Temperatura máxima (°C): ____ | | |
| --- | --- | --- |
| [ ] Hipotermia Temperatura mínima (°C): ____ Data início: ___/___/_____ | | |
| [ ] Dor articular Data início: __/__/____ Extensão: [ ] Oligoarticular [ ] Poliarticular Intensidade: [ ] Leve [ ] Moderada [ ] Intensa  Iniciou aonde?  [ ] Cabeça/pescoço [ ] Tronco/Coluna [ ] Lombossacral [ ] Ombro [ ] Cotovelo [ ] Punho [ ] Dedos das mãos [ ] Dedos dos pés [ ] Planta dos pés [ ] Quadril [ ] Joelhos [ ] Tornozelo [ ] Difuso por todas as articulações | | |
| [ ] Exantema Data início: __/__/____ Duração (dias):___ Tipo exantema: [ ] Pruriginoso [ ] Macular [ ] Maculo-papular | | |
| [ ] Dor abdominal Intensidade: [ ] Leve [ ] Moderada [ ] Intensa | | |
| [ ] Edema de membros Localização: [ ] Articular [ ] Periarticular [ ] Disseminado | | |
| [ ] Rigidez matinal - Se sim, durante quanto tempo: [ ] ≤15dias [ ]15-30 [ ]>30dias | | |
| [ ] Alopecia – Já tinha antes de adoecer? [ ] Sim [ ] Não  - se sim, [ ] aumentou após a doença, quando notou ______________________  [ ] Não notou | | |
| [ ] Depressão – Já tinha antes de adoecer? [ ] Sim [ ] Não , - se sim, [ ] aumentou após a doença [ ] Não notou | | |
| [ ] Prurido | [ ] Artrite | [ ] Calafrios |
| [ ] Cefaleia | [ ] Desânimo | [ ] Petéquias |
| [ ] Dor retroorbitária | [ ] Diarreia | [ ]Equimose |
| [ ] Mialgia | [ ] Náuseas | [ ] Epistaxe |
| [ ] Conjuntivite seca | [ ] Vômitos | [ ] Hematoma |
| [ ] Prostração | [ ] Sonolência | [ ] Irritabilidade |
| [ ] Hipotensão postural | [ ] Lipotimia/Desmaio | [ ] Hepatomegalia |
| [ ] Esplenomegalia | [ ] Coriza | [ ] Tosse |
| [ ] Dispneia | [ ] Dor de garganta | [ ] Faringite |
| [ ] Aftas na boca e/ou garganta | [ ] Paresia | [ ] Paralisia |
| [ ] Apatia | [ ] Linfadenopatia | [ ] Outros, especificar:_________________ |

4.4 HOUVE OUTRAS MANIFESTAÇÕES CLÍNICAS QUE NÃO TINHAM SURGIDO APÓS O QUADRO AGUDO? [ ] Sim [ ] Não [ ] Não informado

- Se Sim, especificar: _______________________

4.5 HOUVE OUTRAS MANIFESTAÇÕES CLÍNICAS QUE SE MANTIVERAM APÓS O QUADRO AGUDO? [ ] Sim [ ] Não [ ] Não informado

- Se Sim, especificar: _______________________

4.6 MANIFESTAÇÕES NEUROLÓGICAS: [ ] Sim [ ] Não [ ] Não informado

- Se sim, quando ___/___/_____, especificar: ^1 – Sim, 2 – Não, 3 – Não informado^

[ ] Meningoencefalite [ ] Encefalite [ ] Convulsões [ ] Paresia [ ] Paralisia [ ] Neuropatia [ ] Síndrome de Guillain-Barré [ ] Síndrome cerebelar [ ] Encefalomielite aguda disseminada [ ] Agitação [ ] Alteração/rebaixamento consciência [ ] Coma [ ] Sinais meníngeos

[ ] Outras, especificar:__________________________________________________

4.7 MANIFESTAÇÕES OCULARES: [ ] Sim [ ] Não [ ] Não informado

- Se sim, quando ___/___/_____, especificar: ^1 – Sim, 2 – Não, 3 – Não informado^

[ ] Neurite optica [ ] Iridiociclite [ ] Episclerite [ ] Retinite [ ] Uveíte [ ] Outras, especificar: _____________________________________________________________

4.8 MANIFESTAÇÕES DERMATOLÓGICAS: [ ] Sim [ ] Não [ ] Não informado

– Se sim, quando ___/___/_____, especificar: ^1 – Sim, 2 – Não, 3 – Não informado^

[ ] Hiperpigmentação fotossensível [ ] Dermatose vesículo-bolhosa [ ] Úlcera aftosa intertriginosa [ ] Isquemia cutânea [ ] Outras, especificar: ______________________

4.9 QUADRO RENAL: [ ] Sim [ ] Não [ ] Não informado

– Se sim, quando ___/___/_____, especificar^: 1 – Sim, 2 – Não, 3 – Não informado^

[ ] Nefrite [ ] Insuficiência Renal Aguda [ ] Redução do debito urinário [ ] Alteração da cor da urina [ ] Outras, especificar: ______________________________

4.10 QUADRO HEMORRÁGICO: [ ] Sim [ ] Não [ ] Não informado

– Se sim, quando ___/___/_____, especificar: ^1 – Sim, 2 – Não, 3 – Não informado^

[ ] Hematemese [ ] Melena [ ] Metrorragia volumosa [ ] Sangramento do SNC [ ] Sangramentos cutâneos [ ] Sangramentos de mucosa oral [ ] Sangramento digestivo alto [ ] Sangramento digestivo baixo [ ] Sangramento cavitário (abdominal, torácico) [ ] Outros, especificar:___________________________________________________

4.11 EVOLUIU PARA CHOQUE: [ ] Sim [ ] Não [ ] Não informado

- Se sim, quando ___/___/_____, especificar: ^1 – Sim, 2 – Não, 3 – Não informado^

[ ] Taquicardia [ ] Pulso débil ou inidentificável [ ] PA diferencial convergente (≤ 20 mmHg) [ ] Extremidades frias [ ] Tempo de enchimento capilar ≥ 3” [ ] Hipotensão arterial (PAS < 90 mmHg) [ ] Outros, especificar: _____________________________

4.12 PRESENÇA DE OUTRAS COMPLICAÇÕES: [ ] Sim [ ] Não [ ] Não informado

- Se sim, quando ___/___/_____, especificar: ^1 – Sim, 2 – Não, 3 – Não informado^ [ ] Miocardite [ ] Discrasias hemorrágicas [ ] Pneumonia [ ] Insuficiência respiratória [ ] Taquidispneia [ ] Hepatite aguda [ ] Pancreatite aguda [ ] Hipoadrenalismo [ ] Icterícia [ ] Edema agudo pulmonar [ ] Infecção associada à assistência à saúde [ ] Outras, especificar: ______________________________________________________

4.13 PROCUROU ATENDIMENTO MÉDICO POR CONTA DESTE QUADRO CLÍNICO? [ ] Sim [ ] Não[ ] Não informado

4.14 SE SIM, QUANTOS SERVIÇOS DE SAÚDE ELE (A) PROCUROU? DESCREVA COMO FOI O ATENDIMENTO:

| **Número** | **Nome serviço saúde** | **Município** | **Data atendimento** | **Hipótese diagnóstica** | **Conduta** | **Soroterapia** | **Quantidade e Data** |
| --- | --- | --- | --- | --- | --- | --- | --- |
|  |  |  | ___/___/_____ |  | [ ] Alta (__/ __/ __)  [ ] Internação  [ ] Transferência | [ ] Sim  [ ] Não | ________ml ___/___/____ ________ml ___/___/____ ________ml ___/___/____  ________ml ___/___/____ |
|  |  |  | ___/___/_____ |  | [ ] Alta (__/ __/ __)  [ ] Internação  [ ] Transferência | [ ] Sim  [ ] Não | ________ml ___/___/____ ________ml ___/___/____ ________ml ___/___/____  ________ml ___/___/____ |
|  |  |  | ___/___/_____ |  | [ ] Alta (__/ __/ __)  [ ] Internação  [ ] Transferência | [ ] Sim  [ ] Não | ________ml ___/___/____ ________ml ___/___/____ ________ml ___/___/____  ________ml ___/___/____ |
|  |  |  | ___/___/_____ |  | [ ] Alta (__/ __/ __)  [ ] Internação  [ ] Transferência | [ ] Sim  [ ] Não | ________ml ___/___/____ ________ml ___/___/____ ________ml ___/___/____  ________ml ___/___/____ |
|  |  |  | ___/___/_____ |  | [ ] Alta (__/ __/ __)  [ ] Internação  [ ] Transferência | [ ] Sim  [ ] Não | ________ml ___/___/____ ________ml ___/___/____ ________ml ___/___/____  ________ml ___/___/____ |

4.15 DURANTE O ATENDIMENTO FEZ USO DE ALGUM MEDICAMENTO? [ ] Sim [ ] Não [ ] Não sabe - Se sim:

| **Nome serviço saúde** | **Classe** | **Medicamento** | **Dose** | **Data** |
| --- | --- | --- | --- | --- |
|  | ( ) Corticóides  ( ) Antiinflamatório ( ) Antibióticos  ( ) Antivirais  ( ) Anticoagulantes ( ) Anticoagulante  ( ) Ansiolítico  ( ) Outro________ |  |  |  |
|  | ( ) Corticóides  ( ) Antiinflamatório ( ) Antibióticos  ( ) Antivirais  ( ) Anticoagulantes ( ) Anticoagulante  ( ) Ansiolítico  ( ) Outro________ |  |  |  |
|  | ( ) Corticóides  ( ) Antiinflamatório ( ) Antibióticos  ( ) Antivirais  ( ) Anticoagulantes ( ) Anticoagulante  ( ) Ansiolítico  ( ) Outro________ |  |  |  |
| **Nome serviço saúde** | **Classe** | **Medicamento** | **Dose** | **Data** |
|  | ( ) Corticóides  ( ) Antiinflamatório ( ) Antibióticos  ( ) Antivirais  ( ) Anticoagulantes ( ) Anticoagulante  ( ) Ansiolítico  ( ) Outro________ |  |  |  |
|  | ( ) Corticóides  ( ) Antiinflamatório ( ) Antibióticos  ( ) Antivirais  ( ) Anticoagulantes ( ) Anticoagulante  ( ) Ansiolítico  ( ) Outro________ |  |  |  |
|  | ( ) Corticóides  ( ) Antiinflamatório ( ) Antibióticos  ( ) Antivirais  ( ) Anticoagulantes ( ) Anticoagulante  ( ) Ansiolítico  ( ) Outro________ |  |  |  |

4.16 DIAGNÓSTICO LABORATORIAL DE ALGUMA DOENÇA INFECCIOSA?

**[ ] Sim [ ] Não – Se sim, qual:**

| **DOENÇA** | **EXAME** | **MATERIAL** | **RESULTADO** | **DADA COLETA** | **DATA DO RESULTADO** |
| --- | --- | --- | --- | --- | --- |
| Chikungunya | IgM | [ ]Sangue [ ]Líquor [ ]Visceras [ ]Não realizado | [ ]Reagente [ ]Não reagente | __/__/_____ | __/__/_____ |
|  | IgG | [ ]Sangue [ ]Líquor [ ]Visceras [ ]Não realizado | [ ]Reagente [ ]Não reagente | __/__/_____ | __/__/_____ |
|  | PCR | [ ]Sangue [ ]Líquor [ ]Visceras [ ]Não realizado | [ ]Reagente [ ]Não reagente | __/__/_____ | __/__/_____ |
|  | Isolamento | [ ]Sangue [ ]Líquor [ ]Visceras [ ]Não realizado | [ ]Reagente [ ]Não reagente | __/__/_____ | __/__/_____ |
| Dengue | IgM | [ ]Sangue [ ]Líquor [ ]Visceras [ ]Não realizado | [ ]Reagente [ ]Não reagente | __/__/_____ | __/__/_____ |
|  | IgG | [ ]Sangue [ ]Líquor [ ]Visceras [ ]Não realizado | [ ]Reagente [ ]Não reagente | __/__/_____ | __/__/_____ |
|  | PCR | [ ]Sangue [ ]Líquor [ ]Visceras [ ]Não realizado | [ ]Reagente [ ]Não reagente | __/__/_____ | __/__/_____ |
|  | Isolamento | [ ]Sangue [ ]Líquor [ ]Visceras [ ]Não realizado | [ ]Reagente [ ]Não reagente | __/__/_____ | __/__/_____ |
| Zika | IgM | [ ]Sangue [ ]Líquor [ ]Visceras [ ]Não realizado | [ ]Reagente [ ]Não reagente | __/__/_____ | __/__/_____ |
|  | IgG | [ ]Sangue [ ]Líquor [ ]Visceras [ ]Não realizado | [ ]Reagente [ ]Não reagente | __/__/_____ | __/__/_____ |
|  | PCR | [ ]Sangue [ ]Líquor [ ]Visceras [ ]Não realizado | [ ]Reagente [ ]Não reagente | __/__/_____ | __/__/_____ |
|  | Isolamento | [ ]Sangue [ ]Líquor [ ]Visceras [ ]Não realizado | [ ]Reagente [ ]Não reagente | __/__/_____ | __/__/_____ |
| outros, especificar______________________________________________ |  |  |  | __/__/_____ | __/__/_____ |
|  |  |  |  |  |  |
|  |  |  |  |  |  |
|  |  |  |  |  |  |
|  |  |  |  |  |  |

4.17 EXAMES LABORATORIAIS

| DATA DA COLETA | __/__/__ | __/__/__ | __/__/__ | __/__/__ | __/__/__ | __/__/__ | __/__/__ |
| --- | --- | --- | --- | --- | --- | --- | --- |
| Hematócrito |  |  |  |  |  |  |  |
| Hemoglobina |  |  |  |  |  |  |  |
| Plaquetas |  |  |  |  |  |  |  |
| Leucócitos |  |  |  |  |  |  |  |
| Neutrófilos |  |  |  |  |  |  |  |
| Eosinófilos |  |  |  |  |  |  |  |
| Basófilos |  |  |  |  |  |  |  |
| Monócitos |  |  |  |  |  |  |  |
| Linfócitos |  |  |  |  |  |  |  |
| Bastonetes |  |  |  |  |  |  |  |
| PCR |  |  |  |  |  |  |  |
| Glicose |  |  |  |  |  |  |  |
| TGO - AST |  |  |  |  |  |  |  |
| TGP - ALT |  |  |  |  |  |  |  |
| Ureia |  |  |  |  |  |  |  |
| Creatinina |  |  |  |  |  |  |  |
| Sódio |  |  |  |  |  |  |  |
| Potássio |  |  |  |  |  |  |  |
| Albumina |  |  |  |  |  |  |  |
| Fosfatase Alcalina |  |  |  |  |  |  |  |
| Bilir. Total |  |  |  |  |  |  |  |
| Bilir. Direta |  |  |  |  |  |  |  |
| Bilir. Indireta |  |  |  |  |  |  |  |
|  |  |  |  |  |  |  |  |
|  |  |  |  |  |  |  |  |

| DATA DA COLETA | __/__/__ | __/__/__ | __/__/__ | __/__/__ | __/__/__ | __/__/__ | __/__/__ |
| --- | --- | --- | --- | --- | --- | --- | --- |
| Glicemia capilar |  |  |  |  |  |  |  |

4.18 REALIZOU PUNÇÃO LÍQUORICA? [ ] Sim [ ] Não **– Se sim, especificar: Data___/___/____** Aspecto: ( ) Límpido ( ) Turvo ( ) Hemorrágico

( )Outro:______________________________________________________________________________________________________________________

| Hemácias (mm³) | Leucócitos (mm³) | Linfócitos (%) | Neutrófilos (%) | Leucócitos (%) | Basófilos (%) | Monócitos (%) | Eosinófilos (%) | Proteína (mg/dl) | Glicose (mg/dl) |
| --- | --- | --- | --- | --- | --- | --- | --- | --- | --- |
|  |  |  |  |  |  |  |  |  |  |

4.19 EXAMES DE IMAGENS

| DATA DA COLETA | __/__/__ | __/__/__ | __/__/__ | __/__/__ | __/__/__ | __/__/__ | __/__/__ |
| --- | --- | --- | --- | --- | --- | --- | --- |
| TC crânio |  |  |  |  |  |  |  |
| RNM |  |  |  |  |  |  |  |
| Raio X |  |  |  |  |  |  |  |
| Ultrassom |  |  |  |  |  |  |  |
| Outros |  |  |  |  |  |  |  |

4.20 HOUVE REMOÇÃO PARA UTI: [ ] Sim [ ] Não

- Se sim, Data admissão: _____/______ /______

Data alta da UTI: _____/______ /______

4.21 EVOLUÇÃO:

[ ] Transferência Data: _____/______ /______

Para onde: _________________________________________

[ ] Alta. Data: _____/______ /______ [ ] Óbito Data: _____/______ /______

4.22 SE ÓBITO, PREENCHA CONFORME A DECLARAÇÃO DE ÓBITO (DO):

A)___________________________________________________________________ B) ____________________________________________________________________ C) __________________________________________________________________

D) ____________________________________________________________________ I) _____________________________________________________________________ II) ____________________________________________________________________

4.23 O CORPO FOI ENCAMINHADO PARA NECROPSIA: [ ] Sim [ ] Não - Se sim, descreva o laudo: __________________________________________________________________________________________________________________________________________________________________________________________________________________________________________________________________________________________________________________________________________________________________________________________________________________________________________________________________________________________________________________________________________________________________________________________________________________________________________________________________________________________________________________________________________________________________________________________________

4.24 SE ÓBITO FETAL OU EM MENORES DE 1 ANO, QUANDO OCORREU EM RELAÇÃO AO PARTO: [ ] Antes [ ] Durante [ ] Após [ ] Ignorado [ ] Não se aplica
