# Supplementary material for: Chikungunya Death Risk Factors in Brazil, in 2017: A case-control study
Source: PLoS One. 2022 Apr 7;17(4):e0260939. doi: 10.1371/journal.pone.0260939 (PMC8989201; doi:10.1371/journal.pone.0260939)
Supplement: S2 File — (DOC) [file pone.0260939.s002.doc]

FACTORS ASSOCIATED WITH DEATHS BY CHIKUNGUNYA: A CONTROL CASE STUDY IN THE CITY OF FORTALEZA, CEARÁ

**Questionnaire N º________**

**CASE [ ] CONTROL [ ]**

**Researcher __________________________________Collection date: _____/_____/______**

**Interviewed: ________________________________________________________________**

**Relationship with case/control: ________________________________________________**

**Contact number:____________________________________________________________**

**Contact address:_____________________________________________________________**

**1. Identification and demographic data**

1.1 NAME:_________________________________________________________________________

1.2 BIRTH DATE: ____/____/_____ 1.3 AGE:_____years 1.3.1 AGE:_____months

1.4 SEX: [ ] Male [ ] Female 1.5 WEIGHT:_______Kg 1.6 HEIGHT:_____cm

1.7 SKIN COLOR: [ ] White [ ] Black [ ] Asian [ ] multiethnic black [ ] Ignored

1.8 SCHOLARITY: [ ] None or less than a year [ ] 1 to 3 years [ ] 4 to 7 years [ ] 8 to 10 years [ ] 11 to 14 years [ ] 15 years or more [ ] Did not answer.

1.9 MARITAL STATUS: [ ] Single [ ] Married [ ] Widow(er) [ ] Divorced [ ] Stable union [ ] Ignored

1.10 THE CASE / CONTROL HAD/HAS ANY RELIGION? [ ] Yes [ ] Not

If yes, which?___________________________________________________________________

1.11 OCCUPATION:_________________________________________________________________

1.12 HOW MANY PEOPLE LIVE IN THE HOUSE?_________(number)

1.13 WHAT IS THE MONTHLY FAMILY INCOME: _________________ REAIS (approximate)

1.14 CURRENT ADDRESS

STATE: ________ COUNTY:_____________________________________________________ STREET/AVENUE:__________________________________________NUMBER:______________DISTRICT: ____________________________________ PHONE: ___________________________

1.15 LIVED AT ANOTHER ADDRESS BEFORE/DURING THE ILLNESS?

[ ] Yes [ ] Not

- If yes:

STATE: _____________________COUNTY:___________________________________________ STREET/AVENUE:__________________________________________NUMBER:______________DISTRICT: ____________________________________ PHONE: ___________________________

1.16 PLACE OF WORK OR ACTIVITY?_________________________________________

1.17 PROBABLE LOCATION OF INFECTION?__________________________________________

1.18 SOMEONE ELSE HAD CHIKUNGUNYA AT HOME? [ ] Yes [ ] Not [ ] Doesn't know

1.19 SOMEONE ELSE HAD CHIKUNGUNYA AT STREET [ ] Yes [ ] Not [ ] Doesn't know

**2. Antecedents**

2.1 DID THE CASE / CONTROL EVER PRESENT A CHANGE IN GLUCOSE LEVEL?

[ ] Yes [ ] Not [ ] Doesn't know

2.2 DID THE CASE / CONTROL HAD OR HAVE HIGH BLOOD PRESSURE?

[ ] Yes [ ] Not [ ] Doesn't know

2.3 DID THE CASE / CONTROL HAVE FIRST DEGREE RELATIVES (parents or siblings or children) with diabetes?

[ ] Yes [ ] Not [ ] Doesn't know – If yes: WHICH?________________________________________

2.4 DID THE CASE / CONTROL HAVE SECOND DEGREE RELATIVES (uncles, aunts, grandparents and first cousins) with diabetes? [ ] Yes [ ] Not [ ] Doesn't know– If yes: WHICH?______________________________

2.5 DOES A DOCTOR OR OTHER HEALTH PROFESSIONAL SAY THAT THE CASE / CONTROL HAVE/HAD ANY OF THESE DISEASES?

[ ] Diabetes [ ] High cholesterol [ ] Angina [ ] heart attack [ ] Stroke [ ] Cardiac insufficiency

[ ] Systemic arterial hypertension [ ] Chronic heart disease [ ] Depression [ ] anxiety [ ] Arthrits [ ] Artrosis [ ] Rheumatic fever [ ] Chronic kidney disease [ ] Chronic Lung Diseases [ ] Asthma [ ] Cancer [ ] Lupus [ ] Neuroleptic disease [ ] Sickle cell anemia [ ] Thalassemia [ ] Dengue

[ ] Zika [ ] Mayaro [ ] Malaria [ ] Leptospirosis

[ ] allergies, specify___________________________________________________

[ ] Other rheumatologic diseases _________________________________________

[ ] Other autoimmune disease _______________________________________________

[ ] Other diseases, specify____________________________________________________

2.6 THE CASE / CONTROL USE A CONTINUOUS MEDICINE?

[ ] Yes [ ] Not [ ] Doesn't know – If yes:

| **DRUG CLASS** | **Specify the drug and dose** | **Start date** | **End date** |
| --- | --- | --- | --- |
| Corticosteroids |  | ____\____\_____ | ____\____\_____ |
| Anti-inflammatory |  | ____\____\_____ | ____\____\_____ |
| Antibiotics |  | ____\____\_____ | ____\____\_____ |
| Antivirals |  | ____\____\_____ | ____\____\_____ |
| Anticoagulants |  | ____\____\_____ | ____\____\_____ |
| Anticonvulsants |  | ____\____\_____ | ____\____\_____ |
| Anxiolytic |  | ____\____\_____ | ____\____\_____ |
| Anti-hipertensivo |  | ____\____\_____ | ____\____\_____ |
| Antihypertensive |  | ____\____\_____ | ____\____\_____ |
| Insulin |  | ____\____\_____ | ____\____\_____ |
| OTHERS |  | ____\____\_____ | ____\____\_____ |

2.7 WAS THERE CLINICAL DECOMPENSATION OF THE CHRONIC DISEASE (for example: need to increase drug dosage)? [ ] Yes [ ] Not [ ] Doesn't know – If yes: WHICH?

____________________________________________________________________________________________________________________________________________________________________________________________________________________________________________________________________________________________________________________________________________________________________________________________________________________________________________________________________________________________________________

**3. Life habits**

**3.1** THE CASE / CONTROL SMOKE / SMOKING CIGARETTES?

[ ] Yes, in the past, but not currently. How long has it been since you stopped?___________________

[ ] Yes, I still smoke. On average how many per day?____________________________________

[ ] Not [ ]

**3.2** DURING THE LAST 12 MONTHS HOW FREQUENCY THE CASE/CONTROL HAS DRINKED AN ALCOHOLIC BEVERAGE?

[ ] Drink/drink daily [ ] Drink/drink 1 to 3 times a week [ ] Drank/drank 4 to 6 times a week

[ ] Drink/drink 1 to 3 times a month [ ] Less than once a month [ ] None [ ] Doesn't know

if yes, how many doses or drinks or beers used/used to have?_______________________

**3.3 PHYSICAL ACTIVITY**

**3.1** DOES THE CASE/CONTROL PRACTICE/PRACT ANY PHYSICAL ACTIVITY?

[ ] Yes [ ] Not [ ] Do not know – If yes: HOW MANY DAYS IN THE WEEK?________________________________________

How long on average did the physical activity last/last?___________________minutes

**4. Vaccination history**

| **Immunobiological** | **Did case/control take a vaccine?** | **Nº Doses** | **Date 1º Dose** | **Date 2º Dose** | **Date 3º Dose** |
| --- | --- | --- | --- | --- | --- |
| Influenza | [ ] Yes [ ] Not  [ ] Do not know |  |  |  |  |
| dT | [ ] Yes [ ] Not  [ ] Do not know |  |  |  |  |
| Dtpa | [ ] Yes [ ] Not  [ ] Do not know |  |  |  |  |
| Hepatitis B | [ ] Yes [ ] Not  [ ] Do not know |  |  |  |  |
| viral triple | [ ] Yes [ ] Not  [ ] Do not know |  |  |  |  |
| yellow fever | [ ] Yes [ ] Not  [ ] Do not know |  |  |  |  |
| Dengue | [ ] Yes [ ] Not  [ ] Do not know |  |  |  |  |

**5. Illness and care history**

4.1 START DATE OF SYMPTOMS:____/____/_______

4.2 What is the first symptom?: [ ] fever [ ] joint pains [ ] rash [ ] back pain [ ] pain in other parts of the body [ ] others____________________________________________________________

4.3 SIGNS AND SYMPTOMS: [1] Yes [2] Not [3] Uninformed

| [ ] Fever start date: ___/___/____ Duration (days):____ Maximum temperature (°C): ____ | | |
| --- | --- | --- |
| [ ] Hypothermia Minimum temperature (°C): ____ start date: ___/___/_____ | | |
| [ ] Joint pain start date: __/__/____ Extension: [ ] few joints [ ] Many joints:  [ ] Light [ ] Moderate [ ] Intense  Started in?  [ ] head/neck [ ] Trunk/Column [ ] lumbosacral [ ] Shoulder [ ] Elbow [ ] Fist [ ] Fingers [ ] toes [ ] feet[ ] hip [ ] Knees [ ] Ankle [ ] All joints | | |
| [ ] Exantema start date: __/__/____ Duration (days):____ type: [ ] itchy rash [ ] Macular [ ] maculopapular rash | | |
| [ ] Abdominal pain Intensity: [ ] Light [ ] Moderate [ ] Intense | | |
| [ ] Limb edema Location: [ ] Articulate [ ] Periarticular [ ] disseminated | | |
| [ ] Morning stiffness - If yes, for how long: [ ] ≤15days [ ]15-30 [ ]>30 days | | |
| [ ] Alopecia – Did you have it before getting sick? [ ] Yes [ ] Not  - if yes, [ ] increased after the illness, when he noticed ______________________  [ ] Doesn´t know | | |
| [ ] Depression – Did you have it before getting sick? [ ] Yes [ ] Not  - if yes, [ ] increased after the illness, when he noticed ______________________  [ ] Doesn´t know | | |
| [ ] Itching | [ ] Arthritis | [ ] Chills |
| [ ] Headache | [ ] Despondency | [ ] Petechiae |
| [ ] Retroorbital pain | [ ] Diarrhea | [ ] Bruise |
| [ ] Myalgia | [ ] Nauseas | [ ] Epistaxis |
| [ ] Dry conjunctivitis | [ ] Vomit | [ ] Bruise |
| [ ] Prostration | [ ] Somnolence | [ ] Irritability |
| [ ] Postural hypotension | [ ] Lipothymia/fainting | [ ] Hepatomegaly |
| [ ] Splenomegaly | [ ] Coryza | [ ] Cough |
| [ ] Dyspnea | [ ] Sore throat | [ ] Pharyngitis |
| [ ] Canker sores in the mouth and/or throat | [ ] Paresis | [ ] Paralysis |
| [ ] Apathy | [ ] Lymphadenopathy | [ ] Others, specify:_________________ |

4.4 OTHER CLINICAL MANIFESTATIONS THAT HAD NOT APPEARED AFTER THE ACUTE PICTURE? [ ] Yes [ ] Not [ ] Doesn't know– If yes: WHICH?______________________________

4.5 THERE WERE OTHER GESTATIONS AFTER THE ACUTE CONDITION? [ ] Yes [ ] Not [ ] Doesn't know

If yes: WHICH?______________________________

4.6 NEUROLOGICAL MANIFESTATIONS: [ ] Yes [ ] Not [ ] Doesn't know

– If yes: Specify:^1 – Yes, 2 – No, 3 – Uninformed^

[ ] Meningoencephalitis [ ] Encephalitis [ ] Seizures [ ] Paresis [ ] Paralysis [ ] Neuropathy [ ] Guillain-Barré Syndrome [ ] Cerebellar syndrome [ ] Acute disseminated encephalomyelitis

[ ] Agitation [ ] Change/downgrade awareness [ ] Coma [ ] Meningeal Signs

[ ] Others, specify:_________________________________________________

4.7 EYE MANIFESTATIONS: [ ] Yes [ ] Not [ ] Doesn't know

– If yes, when ___/___/_____, Specify:^1 – Yes, 2 – No, 3 – Uninformed^

[ ] Optic neuritis [ ] Idiocyclitis [ ] Episcleritis [ ] Retinitis [ ] vUeitis [ ] Others, specify: _____________________________________________________________

4.8 DERMATOLOGICAL MANIFESTATIONS: [ ] Yes [ ] Not [ ] Doesn't know

– If yes, when ___/___/_____, Specify:^1 – Yes, 2 – No, 3 – Uninformed^

[ ] Photosensitive hyperpigmentation [ ] Vesicular bullous dermatosis [ ] Intertriginous aphthous ulcer [ ] Isquemia cutânea [ ] Others, specify:_________________________________________________

4.9 RENAL FRAMEWORK: [ ] Yes [ ] Not [ ] Doesn't know

– If yes, when ___/___/_____, Specify:^1 – Yes, 2 – No, 3 – Uninformed^

[ ] Nephritis [ ] Acute Renal Failure [ ] Reduction of urinary output [ ] Urine color change

[ ] Others, specify:_________________________________________________

4.10 HEMORRHAGIC FRAME: [ ] Yes [ ] Not [ ] Doesn't know

– If yes, when ___/___/_____, Specify:^1 – Yes, 2 – No, 3 – Uninformed^

[ ] Hematemese [ ] Melena [ ] Massive metrorrhagia [ ] CNS bleeding [ ] skin bleeds [ ] Bleeding from oral mucosa [ ] High digestive bleeding [ ] Low digestive bleeding [ ] Cavity bleeding (abdominal, thoracic) [ ] Others, specify:________________________________________

4.11 EVOLVED TO SHOCK: [ ] Yes [ ] Not [ ] Doesn't know

– If yes, when ___/___/_____, Specify:^1 – Yes, 2 – No, 3 – Uninformed^

[ ] Tachycardia [ ] Weak or unidentifiable pulse [ ] Convergent differential PA (≤ 20 mmHg)

[ ] cold ends [ ] Capillary refill time ≥ 3” [ ] hypotension (PAS < 90 mmHg) Others, specify:_________________________________________________

4.12 PRESENCE OF OTHER COMPLICATIONS:

– If yes, when ___/___/_____, Specify:^1 – Yes, 2 – No, 3 – Uninformed^[ ] Yes [ ] Not [ ] Doesn't know

[ ] Myocarditis [ ] Hemorrhagic dyscrasias [ ] Pneumonia [ ] respiratory failure [ ] Tachydyspnea [ ] acute hepatitis [ ] Acute pancreatitis [ ] Hypoadrenalism [ ] Icterus [ ] Acute pulmonary edema [ ] Infection associated with health care

[ ] Others, specify:_________________________________________________

4.13 THE CASE/CONTROL SEEK MEDICAL ATTENTION BECAUSE OF THIS CLINICAL STAFF? [ ] Yes [ ] No [ ] Doesn't know

4.14 IF YES, HOW MANY HEALTH SERVICES DID HE (A) SEEK? DESCRIBE HOW THE SERVICE WAS:

| **Number** | **Health service name** | **County** | **Service date** | **Diagnostic hypothesis** | **Conduct** | **Serum therapy** | **Quantity and Date** |
| --- | --- | --- | --- | --- | --- | --- | --- |
|  |  |  | ___/___/_____ |  | [ ] High (__/ __/ __)  [ ] Hospitalization  [ ] Transfer | [ ] Yes  [ ] Not | ________ml ___/___/____ ________ml ___/___/____ ________ml ___/___/____  ________ml ___/___/____ |
|  |  |  | ___/___/_____ |  | [ ] High (__/ __/ __)  [ ] Hospitalization  [ ] Transfer | [ ] Yes  [ ] Not | ________ml ___/___/____ ________ml ___/___/____ ________ml ___/___/____  ________ml ___/___/____ |
|  |  |  | ___/___/_____ |  | [ ] High (__/ __/ __)  [ ] Hospitalization  [ ] Transfer | [ ] Yes  [ ] Not | ________ml ___/___/____ ________ml ___/___/____ ________ml ___/___/____  ________ml ___/___/____ |
|  |  |  | ___/___/_____ |  | [ ] High (__/ __/ __)  [ ] Hospitalization  [ ] Transfer | [ ] Yes  [ ] Not | ________ml ___/___/____ ________ml ___/___/____ ________ml ___/___/____  ________ml ___/___/____ |
|  |  |  | ___/___/_____ |  | [ ] High (__/ __/ __)  [ ] Hospitalization  [ ] Transfer | [ ] Yes  [ ] Not | ________ml ___/___/____ ________ml ___/___/____ ________ml ___/___/____  ________ml ___/___/____ |

4.15 DID YOU USE ANY DRUG DURING THE SERVICE? [ ] Yes [ ] No [ ] Doesn't know – If yes:

| **Health service name** | **Class Drug** | **medicine** | **Dose** | **Date** |
| --- | --- | --- | --- | --- |
|  | ( ) Corticosteroids  ()Anti-inflammatory  ( ) Antibiotics  ( ) Antivirals  ( ) Anticoagulants  ( ) Anticoagulant  ( ) Anxiolytic  ( ) Other________ |  |  |  |
|  | ( ) Corticosteroids  ()Anti-inflammatory  ( ) Antibiotics  ( ) Antivirals  ( ) Anticoagulants  ( ) Anticoagulant  ( ) Anxiolytic  ( ) Other________ |  |  |  |
|  | ( ) Corticosteroids  ()Anti-inflammatory  ( ) Antibiotics  ( ) Antivirals  ( ) Anticoagulants  ( ) Anticoagulant  ( ) Anxiolytic  ( ) Other________ |  |  |  |
|  |  |  |  |  |
| **Health service name** | **Class Drug** | **medicine** | **Dose** | **Date** |
|  | ( ) Corticosteroids  ()Anti-inflammatory  ( ) Antibiotics  ( ) Antivirals  ( ) Anticoagulants  ( ) Anticoagulant  ( ) Anxiolytic  ( ) Other________ |  |  |  |
|  | ( ) Corticosteroids  ()Anti-inflammatory  ( ) Antibiotics  ( ) Antivirals  ( ) Anticoagulants  ( ) Anticoagulant  ( ) Anxiolytic  ( ) Other________ |  |  |  |
|  | ( ) Corticosteroids  ()Anti-inflammatory  ( ) Antibiotics  ( ) Antivirals  ( ) Anticoagulants  ( ) Anticoagulant  ( ) Anxiolytic  ( ) Other________ |  |  |  |

4.16 LABORATORY DIAGNOSIS OF ANY INFECTIOUS DISEASE?

**[ ] Yes [ ] Not – If yes, witch?**

| **Disease** | **EXAM** | **MATERIAL** | **RESULT** | **GIVEN COLLECTION** | **DATE OF RESULT** |
| --- | --- | --- | --- | --- | --- |
| Chikungunya | IgM | [ ]Blood [ ]Liquor [ ]Viscera [ ]Not performed | [ ]Reagent  [ ]Non-reactive | __/__/_____ | __/__/_____ |
|  | IgG | [ ]Blood [ ]Liquor [ ]Viscera [ ]Not performed | [ ]Reagent  [ ]Non-reactive | __/__/_____ | __/__/_____ |
|  | PCR | [ ]Blood [ ]Liquor [ ]Viscera [ ]Not performed | [ ]Reagent  [ ]Non-reactive | __/__/_____ | __/__/_____ |
|  | Isolation | [ ]Blood [ ]Liquor [ ]Viscera [ ]Not performed | [ ]Reagent  [ ]Non-reactive | __/__/_____ | __/__/_____ |
| Dengue | IgM | [ ]Blood [ ]Liquor [ ]Viscera [ ]Not performed | [ ]Reagent  [ ]Non-reactive | __/__/_____ | __/__/_____ |
|  | IgG | [ ]Blood [ ]Liquor [ ]Viscera [ ]Not performed | [ ]Reagent  [ ]Non-reactive | __/__/_____ | __/__/_____ |
|  | PCR | [ ]Blood [ ]Liquor [ ]Viscera [ ]Not performed | [ ]Reagent  [ ]Non-reactive | __/__/_____ | __/__/_____ |
|  | Isolation | [ ]Blood [ ]Liquor [ ]Viscera [ ]Not performed | [ ]Reagent  [ ]Non-reactive | __/__/_____ | __/__/_____ |
| Zika | IgM | [ ]Blood [ ]Liquor [ ]Viscera [ ]Not performed | [ ]Reagent  [ ]Non-reactive | __/__/_____ | __/__/_____ |
|  | IgG | [ ]Blood [ ]Liquor [ ]Viscera [ ]Not performed | [ ]Reagent  [ ]Non-reactive | __/__/_____ | __/__/_____ |
|  | PCR | [ ]Blood [ ]Liquor [ ]Viscera [ ]Not performed | [ ]Reagent  [ ]Non-reactive | __/__/_____ | __/__/_____ |
|  | Isolation | [ ]Blood [ ]Liquor [ ]Viscera [ ]Not performed | [ ]Reagent  [ ]Non-reactive | __/__/_____ | __/__/_____ |
| others, specify ______________________________________________ |  |  |  | __/__/_____ | __/__/_____ |
|  |  |  |  |  |  |
|  |  |  |  |  |  |
|  |  |  |  |  |  |
|  |  |  |  |  |  |

4.17 LABORATORY EXAMS

| DATE OF COLLECTION | __/__/__ | __/__/__ | __/__/__ | __/__/__ | __/__/__ | __/__/__ | __/__/__ |
| --- | --- | --- | --- | --- | --- | --- | --- |
| Hematocrit |  |  |  |  |  |  |  |
| Hemoglobin |  |  |  |  |  |  |  |
| Platelets |  |  |  |  |  |  |  |
| Leukocytes |  |  |  |  |  |  |  |
| Neutrophils |  |  |  |  |  |  |  |
| Eosinophils |  |  |  |  |  |  |  |
| Basophils |  |  |  |  |  |  |  |
| Monocytes |  |  |  |  |  |  |  |
| Lymphocytes |  |  |  |  |  |  |  |
| Rods |  |  |  |  |  |  |  |
| PCR |  |  |  |  |  |  |  |
| Glucose |  |  |  |  |  |  |  |
| TGO - AST |  |  |  |  |  |  |  |
| TGP - ALT |  |  |  |  |  |  |  |
| Urea |  |  |  |  |  |  |  |
| Creatinine |  |  |  |  |  |  |  |
| Sodium |  |  |  |  |  |  |  |
| Potassium |  |  |  |  |  |  |  |
| Albumin |  |  |  |  |  |  |  |
| Alkaline phosphatase |  |  |  |  |  |  |  |
| Total Bilirubin |  |  |  |  |  |  |  |
| Direct bilirubin |  |  |  |  |  |  |  |
| Indirect bilirubin |  |  |  |  |  |  |  |
|  |  |  |  |  |  |  |  |
|  |  |  |  |  |  |  |  |

| DATE OF COLLECTION | __/__/__ | __/__/__ | __/__/__ | __/__/__ | __/__/__ | __/__/__ | __/__/__ |
| --- | --- | --- | --- | --- | --- | --- | --- |
| Capillary blood glucose |  |  |  |  |  |  |  |

4.18 PERFORMED LIQUOR PUNCTURE? [ ] Yes [ ] Not **– If yes, when Date___/___/____** Aspect: ( ) Clear ( ) Cloudy ( ) hemorrhagic

( )Others:______________________________________________________________________________________________________________________

| Red Cells (mm³) | Leukocytes (mm³) | Lymphocytes (%) | Neutrophils (%) | Basophils (%) | Monocytes (%) | Eosinophils (%) | Protein (mg/dl) | Glucose (mg/dl) |
| --- | --- | --- | --- | --- | --- | --- | --- | --- |
|  |  |  |  |  |  |  |  |  |

4.19 IMAGE EXAMS

| DATE OF COLLECTION | __/__/__ | __/__/__ | __/__/__ | __/__/__ | __/__/__ | __/__/__ | __/__/__ |
| --- | --- | --- | --- | --- | --- | --- | --- |
| Cranial tomography |  |  |  |  |  |  |  |
| Magnetic resonance |  |  |  |  |  |  |  |
| X-ray |  |  |  |  |  |  |  |
| Ultrasound |  |  |  |  |  |  |  |
| Other |  |  |  |  |  |  |  |

4.20 THERE WAS REMOVAL TO ICU: [ ] Yes [ ] Not

- If yes, Admission date:_____/______ /______

ICU discharge date: _____/______ /______

4.21 EVOLUTION:

[ ] Transfer Date: _____/______ /______

where: _________________________________________

[ ] Discharged from hospital. Date: _____/______ /______

[ ] Death Date: _____/______ /______

4.22 IF DEATH, COMPLETE ACCORDING TO THE DEATH CERTIFICATE (DO):

A)___________________________________________________________________ B) ____________________________________________________________________ C) __________________________________________________________________

D) ____________________________________________________________________ I) _____________________________________________________________________ II) ____________________________________________________________________

4.23 THE BODY WAS SENT FOR NECROPSIA:: [ ]Yes[ ] Not

- If yes, describe the report: __________________________________________________________________________________________________________________________________________________________________________________________________________________________________________________________________________________________________________________________________________________________________________________________________________________________________________________________________________________________________________4.24 IF FETAL OR UNDER 1 YEAR OLD, WHEN IT OCCURRED IN RELATION TO DELIVERY: [ ] Before [ ] During [ ] After [ ] Ignored [ ] Not applicable
